# Green valorization of melon and prickly pear byproducts for functional use in minced beef

**DOI:** 10.1038/s41538-026-00922-4

**Published:** 2026-06-10

**Authors:** Nehal Ibrahim, Shaimaa Fayez, Salma H. Katary, Hany Ibrahim, Lamiaa Ibrahim Ahmed, Gehad A. Ezzat

**Affiliations:** 1 Pharmacognosy Department, Faculty of Pharmacy, Ain Shams University, Cairo, Egypt; 2 Faculty of Pharmacy, Ain Shams University, Cairo, Egypt; 3 Pharmaceutical Chemistry Department, Faculty of Pharmacy, Egyptian Russian University, Badr, Cairo, Egypt; 4 Food Hygiene and Control Department, Faculty of Veterinary Medicine, Cairo University, Giza, Egypt

**Keywords:** Biochemistry, Biotechnology, Chemistry, Environmental sciences, Microbiology, Plant sciences

## Abstract

Upcycling agro-waste in sustainable meat preservation supports clean-label standards and SDG12. Herein, we explore an eco-friendly preservation strategy of minced beef which extends product shelf-life and ensures microbial, physicochemical, and sensory integrity. Green extraction methodology of prickly pear peel (PP), melon peel (MP), and melon seeds showed superior greenness and recovery, based on indirect literature comparison with conventional or deep eutectic solvents. In minced beef, MP and PP reduced spoilage indicators (pH, peroxide value, total volatile basic nitrogen), and enhanced the microbial quality in the order of MP > PP > PP + MP. MP significantly reduced staphylococci, *Pseudomonas*, and *Enterobacteriaceae* counts (*P* < 0.05) by 72, 68, and 51%, respectively, compared to the control on day 6. In a viability study, MP completely eradicated *L. monocytogenes* by day 6 of storage. The tested extracts significantly improved sensory attributes in the order of PP > PP + MP > MP compared to the control which spoiled on day 6 (*P* < 0.05). MP showed broad antioxidant spectrum, high total phenolic (98.79 mg GAE/g extract) and flavonoid (59.32 mg QE/g extract) contents, and LC-qTOF-MS/MS phytochemical profile comprising 38 metabolites primarily flavone-*C*-glycosides and fatty acids. This approach warrants further techno-economic analysis to support its transition into a viable circular economy model.

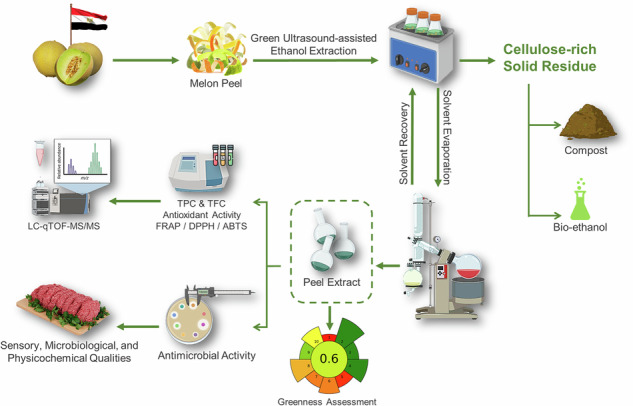

## Introduction

The meat industry is currently challenged to develop innovative preservation strategies that extend product shelf-life, ensure microbial safety and sensory integrity throughout the distribution chain, and meet rigorous clean-label standards^1^. Minced meat is a highly versatile component of the human diet and a suitable base for several meat products like sausages, burgers, meatballs, and sauces. However, its extensive surface area and the excessive handling during processing predispose minced meat to contamination with pathogenic microorganisms such as *Salmonella* species, *Listeria monocytogenes, Escherichia coli*, and *Staphylococcus aureus*^2^. Furthermore, the high-quality nutrients and high moisture content in minced meat make it more susceptible to microbial deterioration and lipid peroxidation with subsequent loss of flavor, texture, nutritional value, and shelf-life^3^. While potent chemical preservatives such as nitrites and benzoates have been employed, they offer no nutritional value and are linked to detrimental health effects, driving increasing consumer concerns^4^. Furthermore, they contaminate wastewater posing substantial risks to ecosystems^5^. Harnessing plant-derived bioactives as functional preservatives is a major focus in sustainable food research to ensure food safety while fulfilling clean-label requirements^6^.

Environmental sustainability has become a global priority in recent years, driven by growing awareness of the need to reduce waste and adopt eco-friendly practices, in alignment with the United Nations Sustainable Development Goals (SDGs)^7^. The agro-food industry generates millions of tons of waste from the cultivation and processing of various crops. According to FAO, about one third of the food produced globally is lost throughout the food supply chain, posing a dual challenge (https://www.fao.org/policy-support/policy-themes/food-loss-and-food-waste/ accessed 20/4/2026). Its high moisture and organic contents promote microbial growth, leading to environmental and health risks and increased greenhouse gas emissions. Meanwhile, this waste represents an underutilized resource, as it is rich in bioactive phytochemicals—principally phenolics—widely recognized for their functional properties^8,9^. To mitigate food losses, artificial intelligence has emerged as a smart tool to enhance food spoilage predictions and optimize supply chain management^10^.

Agro-food waste can be valorized through various routes, including bioenergy production, animal feed, and packaging materials^11^. Additionally, food wastes are increasingly explored as potential food additives and cosmetic ingredients^12,13^. Upcycling agro-food wastes in food industry turns nutrient-rich waste into a resource, provides healthier functional additives, and hence contributes to the achievement of SDG12 (Responsible Consumption and Production) and SDG3 (Good Health and Well-being).

Conventional extraction methods of bioactive compounds (e.g., maceration, infusion, Soxhlet extraction, and hydrodistillation) are limited by their negative environmental impact, long extraction time, and the use of toxic organic solvents. Emerging techniques such as microwave-assisted and pressurized hot water extraction offer better efficiency but often face challenges of scalability, high energy consumption, and degradation of heat-sensitive compounds^14^. More recently, non-thermal approaches—such as ultrasound-assisted extraction (UAE)—have gained attention, particularly when integrated with green solvents, e.g., deep eutectic solvents (DES). This combination can improve extraction efficiency, reduce extraction time, energy consumption, and solvent use, and offer biodegradable, less toxic alternatives aligned with green chemistry principles^15^. However, the high viscosity and complex hydrogen-bonding interactions in DES can reduce ultrasound efficiency. Furthermore, the recovery of target compounds from DES is a major limitation because of their non-volatile nature and high viscosity. The additional steps required to recover the extracted substances are costly and time-consuming and require special instrumentation which may undermine the green nature of DES. Furthermore, the recycling of DES is challenging, increasing overall process complexity and limiting its industrial application^14^.

Bio-alcohols such as bio-ethanol are biodegradable, renewable, minimally toxic, and efficient green solvents for phytochemicals. Ethanol is a versatile, generally recognized as safe (GRAS) solvent. Approved by the FDA for food applications, ethanol is the standard solvent for clean-label products^16^. The generation of bio-ethanol from local agricultural byproducts, its efficient recovery by distillation for further use, and the repurposing of exhausted plant residues in bio-ethanol production^17^ or as a compost ingredient are intelligent approaches in the transformation towards greener practices in food industry^18,19^.

Melon and prickly pear are major summer fruit crops in Egypt. The global production of *Cucumis melo* L., Cucurbitaceae (melon) exceeded 28 Mt in 2024, with Egypt contributing around 485 kt (https://www.fao.org/faostat/en/#data/QCL, accessed 22/4/2026). The consumption of melon generates large quantities of unexploited peel waste which represents 25–44% of the fruit^20^. The resulting wastes rich in phenolics, carotenoids, and oils are a valuable resource, but if unmanaged, they contribute to greenhouse gas emissions, water pollution, and pest problems^21^. Previous data revealed that muskmelon seeds are rich in protein, oil, minerals, amino acids, and fatty acids and that muskmelon seed meal can be used to make biodegradable films^22^. Additionally, 10% replacement of wheat flour with muskmelon seed flour was found to improve the fiber content in bread^23^ and consumer acceptability of cake in terms of taste and texture^24^.

*Opuntia ficus-indica* (L.) Mill. (Cactaceae)—known as prickly pear—is a valuable crop. Due to its adaptability to harsh conditions, prickly pear is well-suited for arid and semi-arid regions and an appreciated alternative crop in sustainable agriculture practices^25^. As a popular low-cost summer treat, the production of prickly pear in Egypt is on the rise, exceeding 31 kt. The thick peels represent significant portion of the fruit weight (40–45%) with approximately 14 kt of peel wastes produced annually^26^. *Opuntia ficus-indica* displays high content of vitamin C, oleic acid, and linoleic acid, valuable amino acid profile, and abundant fiber content, together with functional compounds such as phenolic acids and betalain pigments, encouraging the nutritional, functional, and technological applications of this fruit in food processing^27^.

Although the valorization of agro-food wastes in food preservation aligns with sustainable clean-label standards, critical gaps still exist in improving the greenness of extraction methodology and validating the efficacy of these extracts in complex, highly challenging food systems. The current study aimed to valorize wastes of *Cucumis melo* L. var. *cantalupensis* (melon) and *Opuntia ficus-indica* (L.) Mill. (prickly pear) as functional preservatives for minced beef. A green extraction method was implemented for melon seed and peel and prickly pear peel. The antimicrobial activity of these extracts was evaluated against different pathogenic and spoilage microorganisms. The capacity of these extracts to improve physicochemical quality, maintain sensory integrity, and control microbial spoilage was then assessed in minced beef over a period of 15 days of chilled storage, including a targeted viability study against the resilient, foodborne pathogen *L. monocytogenes*. To link the detected bioactivity with underlying chemical profile, the total phenolic and flavonoid content of the most active extract was quantified, its antioxidant spectrum was evaluated using DPPH, ABTS and FRAP assays, and its phytochemical composition was tentatively characterized by LC-qTOF-MS/MS. A greenness evaluation of the extraction protocol was conducted in comparison with previously established methods. To our knowledge, this is the first comprehensive study to integrate green ethanol extraction of melon and prickly pear peels with practical validation as functional preservatives in minced beef.

## Results and discussion

### Antimicrobial activity and MEC of the selected food waste extracts

Sustainable practices in food industry are gaining significant impetus due to resource scarcity, climate change and global environmental issues. Fruit wastes can be repurposed as cost-effective secondary raw materials for the recovery of bioactive functional ingredients such as phenolics. Integrating these compounds into different food products could extend their shelf-life, improve their microbiological quality, and boost their bioactive profile, nutritional value, and antioxidant capacity while preserving good sensory quality^13^.

In this context, the present study investigated the antimicrobial activities of prickly pear peel (PP), melon peel (MP), and melon seed extracts against a range of bacterial and fungal strains including *S. aureus, E. coli, K. pneumoniae, P. aeruginosa, B. cereus, L. monocytogenes, C. albicans*, and *A. parasiticus* to seek cost-effective sources of functional preservatives. The MEC of the tested fruit wastes, calculated as a percentage, is depicted in Table 1. The current findings showed no difference between the antimicrobial activities of melon peel and seed extracts against all tested microorganisms except for *L. monocytogenes* which was only susceptible to the peel extract. Extracts of melon peels and seeds showed notable activity against *E. coli* with MEC value of 0.1% w/v and to lesser extent against *S. aureus* (MEC = 10%, w/v). Melon peels, likewise, inhibited *L. monocytogenes* in concentration of 5%, w/v. Meanwhile, prickly pear peel showed antimicrobial activity against *S. aureus*, *P. aeruginosa*, and *L. monocytogenes*, with MEC values of 5, 5, and 10% (w/v), producing inhibition zones of 12 ± 0.57, 18.33 ± 0.33, and 17.33 ± 0.33 mm, respectively. The tested strains of *B. cereus, K. pneumoniae, C. albicans*, and *A. parasiticus* appeared to be resistant to all tested extracts.

**Table 1:** The minimum effective concentration, MEC, values and the inhibition zone diameter of food waste extracts on different bacterial and fungal strains

Strains	MEC %, w/v (Inhibition zone diameter, mean ± SD, mm)		
	Prickly peel	Melon peel	Melon seed
*S. aureus*	5 (12.00 ± 0.57)	10 (12.00 ± 0.57)	10 (10.33 ± 0.33)
*E. coli*	NA	0.1 (20.00 ± 0.57)	0.1 (13.00 ± 0.01)
*P. aeruginosa*	5 (18.33 ± 0.33)	NA	NA
*L. monocytogenes*	10 (17.33 ± 0.33)	5 (17.00 ± 0.57)	NA
*B. cereus*	NA	NA	NA
*K. pneumonia*	NA	NA	NA
*C. albicans*	NA	NA	NA
*A. parasiticus*	NA	NA	NA

Our findings agreed to some extent with a previous study showing antibacterial activity of melon seed aqueous extract against *S. aureus* and *P. aeruginosa*^28^. While activity was noticed in our study against *S. aureus*, the ethanol extract of melon seeds did not show any notable activity against *P. aeruginosa* suggesting that different extraction solvents may affect the antimicrobial activity. In another study^29^ reporting on the fruit extract of *Cucumis melo*, good antimicrobial activity was shown against *S. aureus*, *P. aeruginosa* and *C. albicans* with zones of 11, 13 and 16 mm, respectively. However, in the current study, melon peel and seed extracts exhibited no inhibitory activity against either *P. aeruginosa* or *C. albicans*, suggesting that variations in chemical composition among different plant organs may influence their overall biological activity. Contrary to previous reports stating that *S. aureus* was not susceptible to *Cucumis melo* seed ethanol and aqueous extracts^30^, the current study observed antibacterial activity of melon seed ethanol extract (MIC = 10%, w/v).

For prickly pear peels, Ali and coworkers^31^ observed significant antimicrobial properties of their cyclohexanone and ethanol (70% and 90%) extracts against *B. cereus*, *L. monocytogenes*, *S. aureus*, and *E. coli*. However, our findings showed only antibacterial activities against *L. monocytogenes* and *S. aureus*, without any notable effects against *E. coli* or *B. cereus*. Kharrat et al.^32^ showed that the 70% ethanol extract of the whole prickly pear fruit displayed an interesting antimicrobial activity against *B. cereus*, *S. aureus*, *P. aeruginosa*, and *C. albicans*.

Such efficacy against Gram-negative bacterial species is of particular interest since most of the research studies showed that Gram-positive bacteria are more susceptible to plant extracts compared to Gram negative ones^33^. Bioactive compounds, particularly polyphenols such as flavonoids and tannins, derived from plant wastes, contribute to their antimicrobial activity in several ways, including cell membrane breakdown (leading to an increase in cell permeability with loss of cytoplasmic material and disruptions in ion transport), inhibition of biofilm development, and reduction of enzymatic activities and metabolism^34^. Given the observed antibacterial activity of melon and prickly pear peel extracts, future studies should explore their underlying mechanisms of action. Moreover, these findings support further evaluation of their impact on the microbiological, physicochemical, and sensory qualities in minced beef model.

### Microbiological, physicochemical, and sensory qualities of beef minced meat fortified with melon and prickly pear peel extracts

The storage stability of minced beef is significantly impacted by microbial growth, enzymatic degradation, and oxidative rancidity, which are evident by alteration in the sensory attributes of food as well as its pH, PV, and TVB-N. Therefore, these parameters are crucial indicators of meat freshness, lipid oxidation, and protein breakdown, respectively^35^. The incorporation of natural bio-preservatives, particularly fruit and vegetable byproducts, has gained a substantial concern in the modern era as promising alternatives to synthetic preservatives to extend shelf-life through delaying spoilage reactions while enhancing sensory characteristics^36^.

#### Microbiological quality

The effectiveness of melon and prickly pear peel extracts, separately (10%, w/w) and in combination (1:1, 5%, w/w each), in preserving the microbial quality of minced meat was investigated over a period of 15 days of chilled storage compared to the unfortified control group (Table 2). Results revealed deterioration of the control group by the 6th day of storage where the total APC, psychrotrophic, *Pseudomonas*, *Enterobacteriaceae*, staphylococci, and yeast counts reached 7.75 ± 0.03, 7.37 ± 0.02, 7.04 ± 0.03, 7.82 ± 0.02, 5.32 ± 0.02, and 4.49 ± 0.02 log_10_ CFU/g, respectively. However, different treatments affected the tested microbiological parameters with presence of significant differences between the tested treatments and throughout the storage period (*P* < 0.05). Melon peel (MP) was the most effective treatment throughout the storage period, where the total APC, psychrotrophic, *Pseudomonas*, *Enterobacteriaceae*, staphylococci, and yeast counts were 5.06 ± 0.16, 4.95 ± 0.02, 2.3 ± 0.02, 3.88 ± 0.16, 1.6 ± 0.05, and 8.37 ± 0.02 log_10_ CFU/g, respectively, by the end of the storage period. MP-fortified group showed significant difference (*P* < 0.05) compared to the control group (regarding *Enterobacteriaceae*, staphylococci, and yeast counts), and compared to prickly pear peel-fortified group (concerning *Pseudomonas* count (*P* = 0.02) and yeast count (*P* = 0.015)). PP was the second effective treatment followed by the combined group (PP + MP), where the counts were (8.38 ± 0.07, 7.87 ± 0.03), (5.57 ± 0.03, 5.69 ± 0.09), (4.00 ± 0.05, 5.11 ± 0.01), (3.59 ± 0.10, 4.40 ± 0.02), (1.78 ± 0.03, 1.85 ± 0.03), and (7.33 ± 0.02, 8.10 ± 0.05 log_10_ CFU/g) for total APC, psychrotrophic, *Pseudomonas*, *Enterobacteriaceae*, staphylococci, and yeast counts, respectively, with the presence of a significant difference between the counts of *Pseudomonas* (*P* = 0.004), *Enterobacteriaceae* (*P* = 0.002), and staphylococci (*P* < 0.001) of PP treatment and those of the control group. Mold could not be detected in all treatments throughout the entire storage period. Nevertheless, yeast growth reached high counts by the end of the storage time, and neither MP nor PP affected it. Similarly, El-Hawary and coworkers found that prickly pear peel volatile constituents had no effect on the growth of *C. albicans*^37^. Thus, further studies are required to manage the fungal contamination and the resulting deterioration in meat industry.

**Table 2:** Microbial quality of different minced beef groups over a chilled storage period of 15 days. Results are expressed as mean ± SD

Storage period	Treatment	APC	Psychrotrophic	*Pseudomonas*	*Enterobacteriaceae*	Staphylococci	Yeast
		Total counts (log_10_ CFU/g)					
Day 0	C PP MP PP + MP	5.85^abcd^ ± 0.06	4.30^b^ ± 0.14	3.70^bc^ ± 0.09	3.77 ^d^ ± 0.07	4.67^ab^ ± 0.01	3.81 ^f^ ± 0.09
3^rd^ day	C PP MP PP + MP	6.06^abcd^ ± 0.02 4.74 ^d^ ± 0.02 5.47^bcd^ ± 0.02 6.88^abcd^ ± 0.09	4.39^b^ ± 0.02 4.39^b^ ± 0.05 5.86^ab^ ± 0.03 5.33^b^ ± 0.02	4.56^abc^ ± 0.02 3.89^bc^ ± 0.03 2.00^c^ ± 0.05 6.72^ab^ ± 0.02	6.80^ab^ ± 0.09 4.57^bcd^ ± 0.09 3.78 ^d^ ± 0.07 6.20^abc^ ± 0.16	4.60^ab^ ± 0.03 3.67^abc^ ± 0.02 2.09^c^ ± 0.03 5.33^a^ ± 0.02	4.45 ^f^ ± 0.02 4.47 ^f^ ± 0.02 4.30 ^f^ ± 0.02 4.30 ^f^ ± 0.02
6^th^ day	C PP MP PP + MP	7.75^ab^ ± 0.03 5.71^bcd^ ± 0.05 6.82^abcd^ ± 0.02 6.96^abcd^ ± 0.24	7.37^a^ ± 0.02 5.49^ab^ ± 0.03 5.45^b^ ± 0.03 5.53^ab^ ± 0.02	7.04^a^ ± 0.03 4.18^abc^ ± 0.03 2.23^c^ ± 0.07 4.28^abc^ ± 0.03	7.82^a^ ± 0.02 4.95^bcd^ ± 0.03 3.87 ^cd^ ± 0.02 5.60^abcd^ ± 0.09	5.32^a^ ± 0.02 3.31^abc^ ± 0.09 1.50^c^ ± 0.02 4.96^a^ ± 0.02	4.49 ^f^ ± 0.02 4.52 ^f^ ± 0.03 4.60 ^f^ ± 0.03 4.36 ^f^ ± 0.02
9^th^ day	C PP MP PP + MP	Spoiled 6.92^abcd^ ± 0.03 6.12^abcd^ ± 0.07 7.53^abc^ ± 0.03	Spoiled 5.48^ab^ ± 0.03 4.87^b^ ± 0.02 5.48^ab^ ± 0.02	Spoiled 4.78^abc^ ± 0.07 2.65^c^ ± 0.03 4.62^abc^ ± 0.28	Spoiled 4.90^bcd^ ± 0.02 4.82^bcd^ ± 0.02 4.47^bcd^ ± 0.07	Spoiled 3.00^abc^ ± 0.02 1.65^c^ ± 0.05 3.10^abc^ ± 0.14	Spoiled 5.65^e^ ± 0.03 6.94^bcd^ ± 0.03 6.00^de^ ± 0.01
12^th^ day	C PP MP PP + MP	Spoiled 7.26^abcd^ ± 0.09 5.85^abcd^ ± 0.17 7.78^ab^ ± 0.05	Spoiled 5.57^ab^ ± 0.03 4.96^b^ ± 0.03 5.52^ab^ ± 0.02	Spoiled 4.35^abc^ ± 0.05 2.57^c^ ± 0.03 4.79^abc^ ± 0.02	Spoiled 4.60^bcd^ ± 0.31 3.83 ^d^ ± 0.12 4.26 ^cd^ ± 0.24	Spoiled 1.60^c^ ± 0.05 1.63^c^ ± 0.09 2.44^bc^ ± 0.07	Spoiled 6.55^cde^ ± 0.02 7.65^ab^ ± 0.10 7.63^ab^ ± 0.02
15^th^ day	C PP MP PP + MP	Spoiled 8.38^a^ ± 0.07 5.06 ^cd^ ± 0.16 7.87^ab^ ± 0.03	Spoiled 5.57^ab^ ± 0.03 4.95^b^ ± 0.02 5.69^ab^ ± 0.09	Spoiled 4.00^abc^ ± 0.01 2.30^c^ ± 0.02 5.11^abc^ ± 0.01	Spoiled 3.59 ^d^ ± 0.10 3.88 ^cd^ ± 0.16 4.40 ^cd^ ± 0.02	Spoiled 1.78^c^ ± 0.03 1.60^c^ ± 0.05 1.85^c^ ± 0.03	Spoiled 7.33^abc^ ± 0.02 8.37^a^ ± 0.02 8.10^a^ ± 0.05

C: Control group without fortification; PP: Prickly pear peel extract-fortified group; MP: Melon peel extract-fortified group; PP + MP: Combination group of prickly pear peel and melon peel extracts (1:1). ^a-f^: Means with different superscript letters within the same column are significantly different (*P* < 0.05).

Remarkably, all microbiological parameters examined were significantly impacted by the storage duration, except for *Pseudomonas*, which did not significantly change over the course of the storage period (*P* > 0.05) (Table 2). Our findings highlight melon peel extract as a potential antimicrobial agent, compared with other treatments, for preserving minced meat and extending its shelf-life. Previous studies revealed that melon is rich in phenolic compounds (mainly flavonoids)^38^ which have been reported to display antioxidant and antimicrobial activities via disruption of cell membranes, gene regulation, cell–cell communication, and permeability, as well as the interaction with enzymes, and/or deprivation of substrate and metal ions^34^. Prickly pear peel is likewise rich in flavonoids, vitamins, phenolic molecules (ferulic acid derivatives, gallic acid, rutin, and catechin), and betalains which negatively affect the structure, function, and cellular membrane permeability of microorganisms^34,39^. In agreement with the present findings, Palmeri et al reported that prickly pear fruit extract inhibited the growth of several Gram negative and positive strains with efficient reduction of the total mesophilic count, *Enterobacteriaceae*, and *Pseudomonas* during chilled storage of sliced beef, in comparison to the control sample^40^. The present findings partially validate the antibacterial activity observed in Section “Antimicrobial activity and MEC of the selected food waste extracts”. Since MP was the most effective in preserving the bacteriological quality, further investigation of its chemical make-up is warranted.

#### Physicochemical quality

##### pH changes

The physicochemical quality of supplemented chilled minced beef was investigated. The measured pH values of all treatments are illustrated in Table 3. Notably, substantial differences were observed among treatments at day zero (4.96, 5.85, 5.41, and 6.13 for MP, PP, PP + MP, and control, respectively), indicating that the added plant extracts exerted an immediate acidifying intrinsic effect on the minced meat system likely due to their content of organic acids and phenolic compounds. In contrast, an increase in pH values of control was observed during storage, indicating microbial spoilage and accumulation of alkaline nitrogenous compounds with subsequent rapid deterioration of control which became unfit for consumption by the 6th day, according to the EOS (1694/2005) for minced meat. This result aligns with the established spoilage pathways involving psychrotrophic microorganisms such as *Pseudomonas* spp. and *Enterobacteriaceae*, which metabolize amino acids into basic compounds^36^. However, the lower pH values of treated groups observed during storage (*P* < 0.05) cannot be attributed solely to microbial inhibition. Rather, they reflect a combined effect of formulation-induced acidification and storage-related biochemical changes. In similar way, recent studies confirmed that plant phenolics not only inhibit microbial growth but also modify the physicochemical properties of meat systems through direct interactions with proteins and aqueous phases^41^. Again, melon peel extract was the most effective treatment which might be partly attributed to its superior antimicrobial activity against APC, psychrotrophic, *Pseudomonas*, and staphylococci, as illustrated in Table 2.

**Table 3:** Proximate chemical composition (A) and deterioration criteria (B) of the different minced beef groups over a chilled storage period of 15 days. Results are expressed as mean ± SD

A.Proximate chemical composition					
Storage period (day)	Treatment	Moisture %	Protein %	Fat %	Ash %
0	C PP MP PP + MP	72.38^abc^ ± 1.28 72.06^abcd^ ± 0.12 73.05^ab^ ± 0.07 73.32^a^ ± 0.54	20.45^abc^ ± 0.29 20.08^a^ ± 0.03 20.03^c^ ± 0.12 20.06^abc^ ± 0.09	6.19^a^ ± 1.14 5.96^b^ ± 0.16 5.95^b^ ± 0.05 5.93^b^ ± 0.07	1.26^j^ ± 0.28 2.13^hi^ ± 0.14 2.04^i^ ± 0.08 2.32^cdef^ ± 0.02
3	C PP MP PP + MP	72.06^abcd^ ± 1.02 71.48^cde^ ± 0.57 72.44^abc^ ± 1.02 71.83^bcd^ ± 0.86	20.10^ab^ ± 0.12 20.15^abc^ ± 0.05 20.08^abc^ ± 0.07 20.11^abc^ ± 0.05	6.35^a^ ± 0.67 5.14^b^ ± 0.03 5.07^b^ ± 0.07 4.98^b^ ± 0.14	1.33^j^ ± 0.03 2.24^gh^ ± 0.02 2.23^gh^ ± 0.14 2.72^ab^ ± 0.03
6	C PP MP PP + MP	72.06^bcd^ ± 1.02 71.09^cde^ ± 0.86 71.82^bcd^ ± 0.52 70.90^de^ ± 0.85	20.10^abc^ ± 0.17 20.21^abc^ ± 0.22 20.09^abc^ ± 0.07 20.10^abc^ ± 0.03	6.35^a^ ± 0.67 5.13^b^ ± 0.05 5.07^b^ ± 0.61 5.21^b^ ± 0.06	1.33^j^ ± 0.03 2.30 ^g^ ± 0.01 2.30 ^g^ ± 0.09 2.77^ab^ ± 0.07
12	C PP MP PP + MP	Spoiled 68.02^ghi^ ± 1.11 69.11^fgh^ ± 0.48 67.88^ghi^ ± 1.33	Spoiled 20.09^abc^ ± 0.14 20.07^abc^ ± 0.06 20.05^abc^ ± 0.09	Spoiled 4.97^b^ ± 0.02 5.13^b^ ± 0.02 5.25b ± 0.03	Spoiled 2.39^defg^ ± 0.01 2.54 ^cd^ ± 0.02 2.87^a^ ± 0.02
15	C PP MP PP + MP	Spoiled 66.97^i^ ± 1.68 68.11^ghi^ ± 0.09 67.64^hi^ ± 1.40	Spoiled 20.06^abc^ ± 0.07 20.03^abc^ ± 0.05 20.00^bc^ ± 0.09	Spoiled 5.01^b^ ± 0.07 5.05^b^ ± 0.03 5.00^b^ ± 0.05	Spoiled 2.62^cde^ ± 0.01 2.69^bc^ ± 0.01 2.74^a^ ± 0.10
**B.Deterioration criteria**					
**Storage period (day)**	**Treatment**		**pH**	**PV (meq/kg)**	**TVB-N (mg/100** **g)**
0	C PP MP PP + MP		6.13^b^ ± 0.01 5.85 d ± 0.02 4.96^i^ ± 0.02 5.41^e^ ± 0.01	4.59^hi^ ± 0.01 4.47^hi^ ± 0.02 4.32^hi^ ± 0.03 4.30^hi^ ± 0.04	6.69^i^ ± 0.02 6.54^i^ ± 0.01 6.51^i^ ± 0.01 6.56^i^ ± 0.03
3	C PP MP PP + MP		6.24^b^ ± 0.07 5.65^c^ ± 0.01 4.92^ij^ ± 0.02 5.67^e^ ± 0.03	6.66^b^ ± 0.33 3.51^k^ ± 0.03 3.45^k^ ± 0.01 3.38^k^ ± 0.07	8.82^b^ ± 0.05 5.26 ^h^ ± 0.12 5.20 ^h^ ± 0.43 6.01^ef^ ± 0.16
6	C PP MP PP + MP		6.53^a^ ± 0.02 4.85^jk^ ± 0.01 4.81^ij^ ± 0.05 5.79 ^f^ ± 0.01	20.84^a^ ± 0.19 4.14^j^ ± 0.02 4.10^j^ ± 0.07 4.02^j^ ± 0.02	16.36^a^ ± 0.33 5.56^efg^ ± 0.02 5.40^gh^ ± 0.21 6.45 ^h^ ± 0.09
9	C PP MP PP + MP		Spoiled 5.28^i^ ± 0.01 5.13^fgh^ ± 0.02 5.92^ij^ ± 0.02	Spoiled 5.43^def^ ± 0.02 5.44 ^d^ ± 0.03 5.09^efg^ ± 0.09	Spoiled 6.61^c^ ± 0.03 5.54^c^ ± 0.07 5.26 ^h^ ± 0.03
12	C PP MP PP + MP		Spoiled 5.72^k^ ± 0.02 5.16 ^h^ ± 0.10 5.95 ^h^ ± 0.12	Spoiled 5.45 ^d^ ± 0.02 5.55 ^cd^ ± 0.02 5.35^de^ ± 0.09	Spoiled 6.74^c^ ± 0.03 5.62^e^ ± 0.03 5.63^fgh^ ± 0.01
15	C PP MP PP + MP		Spoiled 5.97^ijk^ ± 0.02 5.32^gh^ ± 0.01 6.13^fgh^ ± 0.02	Spoiled 6.46^c^ ± 0.48 6.67^b^ ± 0.01 5.57 ^d^ ± 0.02	Spoiled 6.38^c^ ± 0.02 6.35 ^d^ ± 0.07 7.95 ^d^ ± 0.01

C: Control group without fortification; PP: Prickly pear peel extract-fortified group; MP: Melon peel extract-fortified group; PP + MP: Combination group of prickly pear peel and melon peel extracts (1:1). ^a-k^:Means with different superscript letter within the same column are significantly different (*P* < 0.05).

Similar trends were reported by Murshed et al. who observed that meat supplemented with orange peel extract maintained a lower pH during 15 days of chilled storage compared to controls due to the antimicrobial effect of phenolic compounds^42^. Previous studies demonstrated that natural fruit peel extracts reduced the microbial metabolic activity, thereby retarding pH increase in meat^35^. Nevertheless, Sharma et al found that peel extracts with low acidity or buffering capacity had a limited impact on pH control, further supporting the multifactorial nature of pH modulation^43^.

#### Lipid oxidation (peroxide value)

Peroxide value, a crucial marker of early lipid degradation, was estimated and the findings are shown in Table 3. Throughout the storage period, PV increased gradually in all treatments, reflecting the inherent susceptibility of minced meat to oxidative deterioration due to its disrupted muscle structure and exposure to pro-oxidants such as heme iron^6^. The control sample exhibited the highest PV; 10.74 meq/kg on day 6 which exceeded the permissible limits guided by EOS (1694/2005). On the other hand, PP, MP, and PP + MP maintained significantly lower PV levels (4–4.5 meq/kg till day 6), probably due to the antioxidant activity of their phenolic content. The most prominent effect was observed in the combination group (PV = 5.56 meq/kg on day 15) which can be attributed to synergistic interaction, as formerly reported for multi-extract blends in meat systems^44^. The reduction in PV may be attributed in part to the antioxidant activity of plant-derived phenolics, which act through hydrogen atom donation and metal chelation, thereby interrupting free radical chain reactions and delay lipid oxidation^45^.

These findings are supported by Abbas et al. who recorded good DPPH radical-scavenging activity (IC₅₀ = 38.56 µg/ml) in *Cucumis melo* peel extract, comparable to butylhydroxytoluene (BHT)^46^, and Parafati et al. who reported lower lipid oxidation parameters in beef burger treated with *Opuntia ficus-indica* extract^47^. In contrast, Pateiro and coworkers reported that the antioxidant activity of plant extracts declines due to the high-fat content and higher storage temperature^48^. However, it is important to recognize that plant extracts may also influence PV measurements through interactions with lipid oxidation intermediates or by modifying the physicochemical environment of the meat matrix which should be taken into consideration while measuring PV.

#### Protein degradation

TVB-N is a prominent indicator of incipient degradation in meat protein caused by proteolytic microbial activities. TVB-N gradually increases with the prolongation of meat storage period and its elevation is directly accompanied by high microbial count and alteration in sensory acceptability^49^. The results (compiled in Table 3) show a progressive increase in TVB-N in all treatments during chilled storage, with the control sample exceeding acceptable limits by day 6 (16.35 mg/100 g). In contrast, PP, MP, and their combination maintained significantly lower TVB-N values, which were within the acceptable thresholds set by EOS (1694/2005) till day 15, suggesting improved protein stability. MP resulted in the lowest TVB-N values, suggesting potential inhibition of proteolytic bacterial degradation. These results support our data of microbiological quality (Table 2) and pH changes (Table 3) where melon peel treatment displayed the best microbiological quality and can hence prevent the microbial-linked increase in pH and proteolysis.

The lower TVB-N values in treated samples may be partially attributed to the antimicrobial effects of plant-derived phenolics, which can inhibit spoilage microorganisms and associated deamination processes^3^. Furthermore, phenolic compounds are known to interact with muscle proteins through hydrogen bonding and hydrophobic interactions, potentially enhancing protein stability and limiting the release of volatile nitrogenous compounds^50^. However, it is also important to consider that the incorporation of plant extracts may influence TVB-N measurements through matrix modification, dilution effects, or interactions with analytical procedures. Therefore, the observed reductions in TVB-N may be attributed to a combination of antimicrobial activity, protein stabilization, and formulation-related influences.

Ghoneim and coworkers ‏demonstrated that natural plant extracts inhibited TVB-N accumulation in minced meat during cold storage^51^. Another study confirmed similar TVB-N steadiness in fruit peel polyphenols-treated groups^52^. Lower TVB-N values were recorded in meat products supplemented with onion peel, potato peel, and their combination, as well as mango peel eggplant combination during storage period which led to shelf-life extension^49^.

#### Proximate chemical composition

The analyses of moisture, protein, fat, and ash content (Table 3) revealed a stable pattern during storage period for all supplemented and control groups up to day 6 with changes thereafter. The significant reduction in moisture content from 72% at the beginning of the storage period to 67% at the end of the experiment can be attributed to progressive drip loss and protein denaturation, leading to a decline in water-holding capacity during refrigerated storage. Such moisture loss is a typical postmortem change and is known to influence microbial activity, sensory attributes, and can reflect on the concentration of other components such as protein and ash contents^53^. Reduction in moisture may slightly limit microbial proliferation due to decreased water availability; however, under chilled storage conditions, this effect is generally insufficient to account for the observed differences between treatments. The lower microbial counts in supplemented samples are therefore more likely associated with the antimicrobial activity of bioactive compounds present in plant extracts rather than moisture reduction alone^54^.

The reduced moisture content throughout the chilled storage period also increased the firmness and reduced the juiciness, which negatively affected texture and overall acceptability scores in control samples during extended storage. Furthermore, the observed low tenderness and juiciness scores in PP-supplemented group at the end of storage could be associated with the reduced moisture content. In addition, the significant elevation in ash contents of combination group (2.74%), MP group (2.69%), and PP group (2.62%) may be attributed to the normal dehydration occurring during storage and the higher mineral contents in plant tissues^55^. Protein and fat contents showed minimal variation throughout the storage period. Protein and fat content scores were (20.45, 6.19%); (20.08, 5.96%); (20.03, 5.95%), and (20.06, 5.93%) in control, PP, MP, PP + MP, respectively, at day zero compared to (20.06, 5.01%); (20.03, 5.05%), and (20.00, 5.00%) in PP, MP, PP + MP, respectively, by the end of the storage period indicating that the extracts did not substantially alter the basic chemical composition of the fortified minced beef groups (*P* > 0.05) and throughout the storage period (*P* > 0.05). These findings suggested that the major effect of the extracts was functional rather than compositional, enhancing product stability without compromising nutritional attributes. Similar patterns were reported by Pateiro and coworkers, who observed enhanced oxidative stability of minced meat due to the incorporation of phenolic-rich peel powders without impacting the meat chemical composition^48^.

#### Sensory attributes

**Fig. 1:**
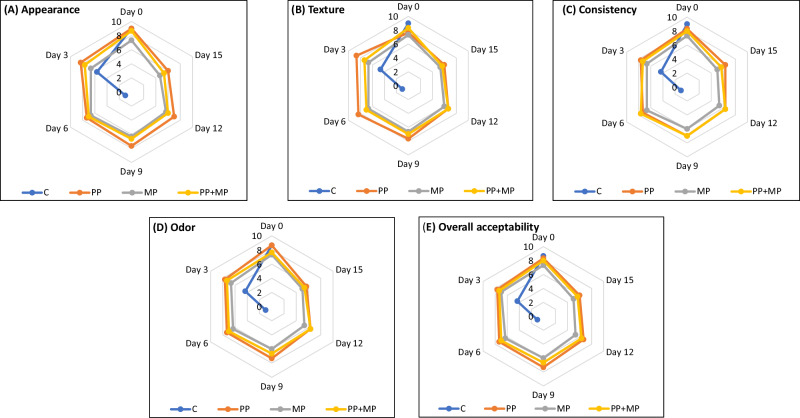
Sensory quality of the fortified raw minced beef over a chilled storage period of 15 days. (**A**): Appearance, (**B**): Texture, (**C**): Consistency, (**D**): Odor, (**E**): Overall acceptability; C: control group, PP: minced beef fortified with prickly pear peel extract (10%, w/w), MP: minced beef fortified with melon peel extract (10%, w/w), PP + MP: minced beef fortified with a combination of prickly pear and melon peel extracts (1:1, 5% w/w each).

Results of the sensory evaluation are shown in Fig. 1. For both raw and cooked samples, no significant differences (*P* > 0.05) were observed at day zero among all treatments for most sensory attributes, indicating that the addition of PP, MP, and their combination did not adversely affect the initial sensory quality. However, as storage progressed, the control samples showed a rapid and significant decline in all sensory parameters, becoming unacceptable by the 6^th^ day of refrigerated storage, likely due to associated microbial spoilage and oxidative deterioration^56^. In contrast, the raw treated samples exhibited a prominent sensory stability, particularly PP-fortified group which consistently recorded the highest scores for appearance, odor, texture, and overall acceptability across the storage period, followed by the combined treatment (PP + MP). Nevertheless, MP showed comparatively lower scores particularly for appearance and odor which might be attributed to its relatively high carbohydrate content^57^ resulting in darker and stickier minced beef compared to PP- and combination-fortified groups. However, the overall sensory scores of MP group did not exceed the acceptable limits till the end of the storage period. The improved sensory stability in treated samples may be attributed to the protective role of phenolic compounds in stabilizing muscle proteins and limiting oxidative and microbial deterioration. This effect likely contributed to better retention of structural integrity, improved textural perception, and preserving color and odor^58^.

**Fig. 2:**
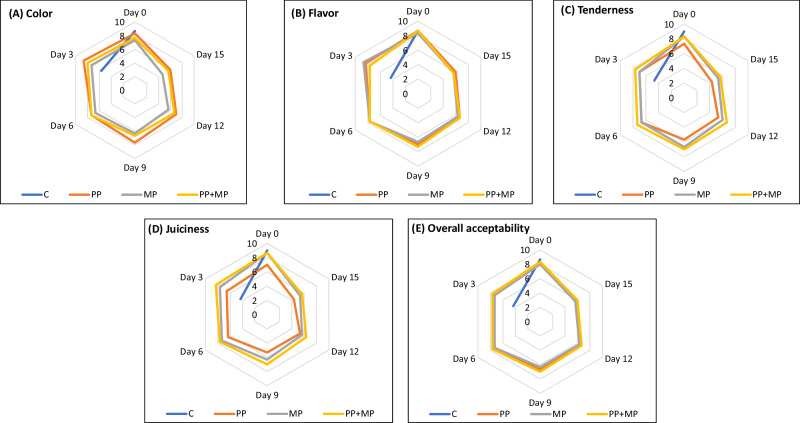
Sensory quality of the fortified cooked minced beef over a storage period of 15 days. (**A**): Color, (**B**): Flavor, (**C**): Tenderness, (**D**): Juiciness, (**E**): Overall acceptability; C: control group, PP: minced beef fortified with prickly pear peel extract (10%, w/w), MP: minced beef fortified with melon peel extract (10%, w/w), PP + MP: minced beef fortified with a combination of prickly pear and melon peel extracts (1:1, 5% w/w each).

Regarding the sensory scores of the cooked samples (Fig. 2), treated samples maintained significantly higher (*P* < 0.05) sensory scores than the control, with the slowest decline observed throughout the storage. Among treatments, PP consistently recorded the highest values for color and flavor, while MP and PP + MP showed comparable or slightly higher values for tenderness and juiciness at certain storage periods. On the 15th day, PP + MP treatment maintained overall acceptability scores around 6, which were higher than MP. The combined treatment exhibited performance comparable to PP in most attributes and showed relatively better retention of tenderness and juiciness at later storage stages, indicating a partial synergistic effect, likely due to the combined action of different phenolic compounds^59^. The consistency between raw and cooked sensory trends indicated that the same deterioration mechanisms operate in both states. However, cooked attributes, such as flavor and juiciness, were more sensitive to storage, reflecting the cumulative effects of lipid oxidation and protein denaturation during cooking. Odor (raw) and flavor (cooked) were particularly affected by storage, emphasizing their importance as primary indicators of spoilage. Similarly, texture (raw) and tenderness (cooked) declined significantly due to protein breakdown^60^.

The significant interaction between treatment and storage period indicated that the effectiveness of treatments depended on storage duration. While differences among treatments were minimal at the initial stage, they became more pronounced over time, particularly PP and PP + MP which showed superior performance at extended storage periods (10–15 days). Research on the use of prickly pear and melon peel extracts in minced meat preservation and fortification is limited, and their application in other food categories has been scarcely investigated. For instance, Hussain et al. reported that a biscuit supplemented with 5% melon peel powder exhibited significantly higher sensory scores (*P* < 0.05)^61^. Furthermore, Zor et al. demonstrated that fortification of meatballs with various vegetable peels had positive impact on their sensory attributes. They attributed the stable sensory scores for color and odor to the antioxidant activity of phenolic compounds^62^. Future studies are warranted to investigate the influence of such extracts on meat products in larger sensory panel size with descriptive sensory analysis and instrumental measurements to provide a more comprehensive assessment. Additionally, future research should focus on strategies that enhance efficacy at lower doses, e.g., the application of hurdle technology and nano-encapsulation approaches.

### Viability of *L. monocytogenes* in minced beef fortified with melon and prickly pear peel extracts

**Fig. 3:**
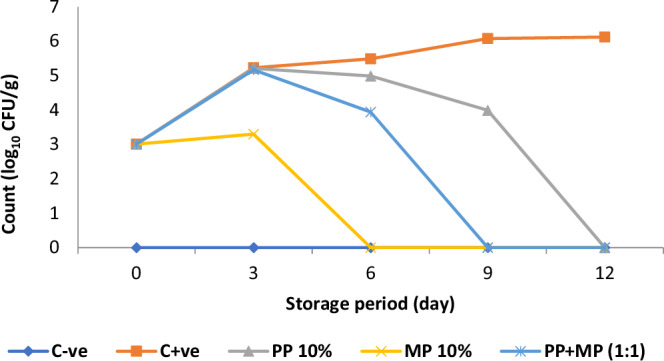
Influence of melon peel and prickly pear peel extracts on the viability of *L.* *monocytogenes* in minced beef over a period of 15 days. C-ve: negative control group, C+ve: positive control group, PP: minced beef supplemented with prickly pear peal extract (10%, w/w), MP: minced beef supplemented with melon peal extract (10%, w/w), PP + MP: minced beef supplemented with a combination of prickly pear and melon peal extracts (1:1, 5% w/w each).

The effectiveness of MP and PP extracts in controlling the viability of *L. monocytogenes* as one of the foodborne pathogens in minced beef (inoculated with 3.01 log_10_ CFU/g) was investigated over a period of 15 days. Extracts were applied separately at a 10%, w/w concentration and in a combined (1:1) regimen (5%, w/w each). *L. monocytogenes* was totally absent in the negative control sample throughout the storage period, confirming no inherent contamination. Meanwhile, it completely disappeared by the end of the 6th day of storage in MP-fortified minced beef, compared to the 12^th^ and 9th days for PP and the mixed treatment, respectively (*P* < 0.05), as evidenced by the absence of growth on the surface of Palcam agar plates (~less than 10 CFU/g) (Fig. 3). In contrast, the positive control group (inoculated minced beef without fortification) continued to harbor *L. monocytogenes* till the end of the storage period, exceeding a count of 6 log_10_ CFU/g with 3-log increase (*P* < 0.05).

Many flavonoids previously identified in melon have been reported to exert antilisterial effects through various mechanisms^38^. For example, apigenin inhibits the activity of DNA gyrase and hydroxy acyl-acyl carrier protein dehydratase^63^. Luteolin interferes with the production of listeriolysin O, one of the virulence factors of *L. monocytogenes*^64^. Additionally, previous studies reported that flavonoids can inhibit the biofilm formation of *L. monocytogenes*, with inhibition percentage ranging from 2 to 100%^65^. *O. ficus-indica* extract was shown to reduce the growth of *L. monocytogenes* on fresh-cut apples to undetectable levels^66^.

The acidifying effect of extracts should be taken into consideration since supplementation of minced meat resulted in significantly lower pH values in MP (4.96), PP (5.85), and PP + MP (5.41) compared to the control (6.13) (*P* < 0.05), as shown in Table 2. Acidification of minced meat represents an unfavorable and stressful condition for microbial growth^41^.

These results support those obtained in Section “Antimicrobial activity and MEC of the selected food waste extracts”, where MP extract displayed the best inhibition of *L. monocytogenes*. This promising antilisterial activity of MP extract, compared to prickly pear peel and the combined treatment, encourages its potential use as a sustainable food preservative to enhance the safety and bacteriological quality of minced beef. Nevertheless, further research is warranted to unveil the phytochemical composition of melon peel extract (which is carried out in Section 2.6).

### Antioxidant activity

To explore whether melon peel extract display antioxidant capacity (hence confirm its ability to improve the oxidative stability and shelf-life of minced meat), the antioxidant activity was assessed by different in vitro assays; DPPH, ABTS, and FRAP. These assays cover the two major antioxidant mechanisms; single electron transfer (SET) and hydrogen atom transfer (HAT). FRAP assay is purely a SET-based reaction, while DPPH and ABTS assays are mixed mode reactions (SET and HAT)^67^.

MP extract showed a DPPH radical-scavenging IC_50_ of 23.62 ± 0.87 µg/mL compared to ascorbic acid (IC_50_ = 10.19 ± 0.54 µg/mL). Previous studies highlighted the potent DPPH scavenging potential of *C. melo* peel methanol extracts with IC_50_ of 38.56 and 4.61 µg/mL, which were comparable to the standard antioxidant BHT^46,68^. On the contrary, Morais et al reported much less DPPH scavenging activity for raw melon peel (IC_50_ of 458.60 µg/mL)^69^. Additionally, MP extract exhibited ABTS radical-scavenging IC_50_ of 14.75 ± 0.54 µg/mL, compared to ascorbic acid (IC_50_ = 10.65 ± 0.89 µg/mL). In a previous study, *C. melo* var. *agrestis* showed higher ABTS scavenging activity (IC_50_ = 7.66 µg/mL), almost equipotent to quercetin (IC_50_ = 6.50 µg/mL)^70^. Despite the presence of various classes of natural antioxidants (e.g., carotenoids, organosulfur compounds, unsaturated fatty acids, and vitamins), polyphenolic compounds such as flavonoids are the most powerful dietary antioxidants. Their radical-scavenging potential could be ascribed to their hydrogen-donating ability, where they can directly neutralize free radicals, resulting in the formation of less reactive aromatic radical species stabilized by electron delocalization. The number and position of phenolic hydroxyl groups on the flavonoid backbone are the major determinant of their intrinsic radical-scavenging activity, with structures containing ortho-dihydroxy groups being the most efficient radical scavenger^71^. Furthermore, the reducing power of plant extracts is considered an index of their antioxidant capacity^67^. The reducing power of MP extract was assessed by FRAP assay, showing an IC_50_ of 43.98 ± 1.69 µg/mL compared to ascorbic acid (IC_50_ = 20.88 ± 0.76 µg/mL).

The DPPH and ABTS assay results reflect the extract ability to scavenge lipid radicals by hydrogen atom donation, thereby terminating lipid peroxidation chain reactions. Meanwhile, the FRAP assay illustrates its capacity to reduce transition metals, thus inhibiting metal-catalyzed oxidation pathways. While in vitro antioxidant assays cannot predict in situ performance due to complex matrix effects, the significant reduction in PV in fortified minced beef (Table 3) is central, as it confirms the antioxidant activity in the complex meat matrix. Together, these findings encourage the valorization of melon peel extract as a functional additive to protect minced meat from oxidative deterioration.

### Total phenolic (TPC) and total flavonoid contents (TFC) of melon peel extract

Phenolic compounds are among the most common plant secondary metabolites which are crucial for plant defense against different stresses. Their redox properties are attributed to the presence of one or more phenolic hydroxyl groups that enable them to display antioxidant effects and contribute to the antimicrobial activity by acting as proton exchangers, leading to depolarization of microbial cell membranes and disruption of membrane integrity^72^. The TPC in melon peel ethanol extract was found to be 98.79 ± 1.21 mg GAE/g dry extract (2.72 ± 0.03 mg GAE/g fresh peel), whereas its TFC was estimated as 59.32 ± 1.08 mg QE/g dry extract (1.63 ± 0.03 mg QE/g fresh peel). Sroy et al reported slightly lower TPC in the methanol peel extract of *C. melo* L. var. *reticulatus* (2.45 ± 0.24 mg GAE/g fresh peel)^73^.

While conventional extraction of *C. melo* L. (*maazoun* cultivar) peels recovered phenolic compounds at 3.32 mg GAE/g extract^74^, UAE with 95% ethanol resulted in a substantial 8-fold higher TPC (25.48 mg GAE/g extract) as reported by Vella et al^75^. The TPC can vary considerably according to sample treatment (e.g., drying) and extraction conditions (e.g., technique, solvent, extraction time and temperature, and sample to solvent ratio). Several other factors include genotype, geographical origin, environmental factors, degree of ripening, and postharvest conditions^75^. Another study comparing the peels of muskmelon (*Cucumis melo* var. *cantaloupe*), canary melon (*Cucumis melo* var. *fonzy*), and watermelon (*Citrullus lanatus*, var. *augusta*) showed muskmelon hydroethanolic extract to display the highest TPC (167.49 ± 0.04 mg GAE/100 g dry peel), TFC (79.16 ± 0.05 mg QE/100 g), and total antioxidant activity (56.92 ± 0.05 mg Trolox equivalent/100 g)^61^. Therefore, the observed antioxidant properties of melon peels are probably attributed to their high TPC and TFC. These findings warrant an in-depth phytochemical investigation to fully characterize the composition of the MP extract.

### LC-qTOF-MS/MS chemical profiling of melon peel extract

**Fig. 4:**
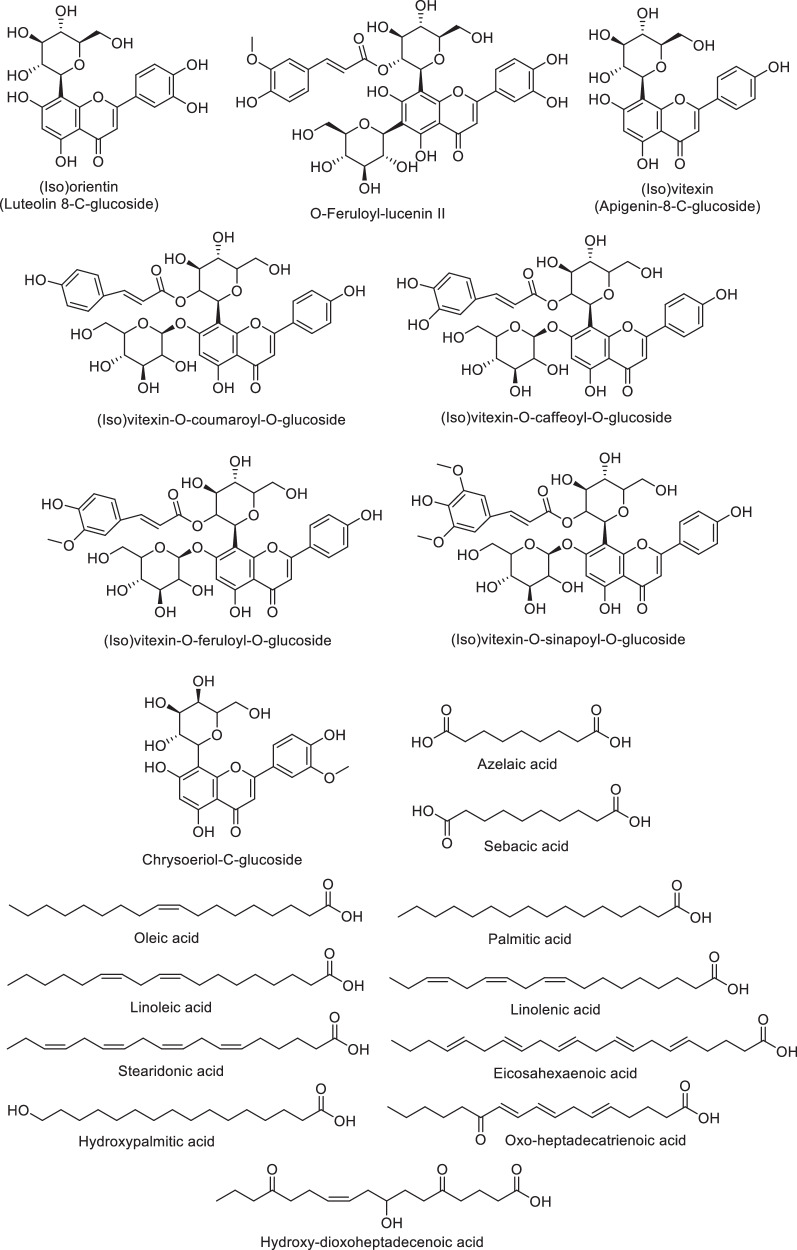
Chemical structures of some of the tentatively identified flavonoids and fatty acids in melon peel extract.

A total of 38 compounds were tentatively identified in melon peel ethanol extract based on comparison of observed R_t_, accurate molecular masses, predicted molecular formulae, and MS/MS spectra with literature and databases. The tentatively identified metabolites were classified into 14 flavonoids, 15 fatty acids, 2 organic acids, 1 megastigmane, and 6 miscellaneous metabolites as listed in Table 4 and Fig. 4.

**Table 4:** Dereplication of metabolites in *C. melo* L. var. *cantalupensis* peel extract using LC-qTOF-MS/MS analysis in negative and positive ion modes

Peak no.	t_R_ (min)	*m/z* ^*-*^	Annotation	MS/MS	Molecular formula	∆ (ppm)	Chemical class	References
1.	0.86	191.0195	Citric acid	111, 103	C_6_H_8_O_7_	1.69	Organic acids	^111^
2.	0.91	290.0874	*N*-hexosyl pyroglutamate	200, 170, 128	C_11_H_17_NO_8_	−0.66	Amino acid derivative	^38^
3.	1.46	179.0552	Hexose	118, 101	C_6_H_12_O_6_	−2.03	Sugars	^112^
4.	6.77	165.0541	Phenyllactic acid	147, 119, 103	C_9_H_10_O_3_	−6.48	Organic acids	^38^
5.	7.17	609.1462	Luteolin-di-*C*-hexoside (lucenin-II)	---	C27H30O16	1.05	Flavonoids	
6.	7.46	447.0938	Luteolin-*C*-hexoside (iso/orientin)	429, 357, 327, 285	C_21_H_20_O_11_	2.38		
7.	8.01	593.1538	Isovitexin-*O*-hexoside (meloside A)	593, 431, 413, 311, 293	C_27_H_30_O_15_	5.32		^38,76^
8.	8.27	755.1842	Iso/orientin -*O*-(*O*-coumaroyl) hexoside	755, 635, 609, 429	C_36_H_36_O_18_	2.46		^113^
9.	8.28	623.1617	Lucenin-II-methyl ether	---	C_28_H_32_O_16_	0.79		^111^
10.	8.31	785.1936	*O*-Feruloyl-lucenin-Ⅱ	665, 609, 575, 545, 489	C_37_H_38_O_19_	0.91		^38^
11.	8.32	431.0990	Apigenin-*C*-hexoside (iso/vitexin)	---	C_21_H_20_O_10_	2.73		^76^
12.	8.66	461.1085	Chrysoeriol-*C*-hexoside (iso/scoparin)	425, 371, 343, 341	C_22_H_22_O_11_	0.56		^114^
13.	8.68	755.1838	Iso/vitexin-*O*-(*O*-caffeoyl) hexoside	---	C_36_H_36_O_18_	1.93		^38^
14.	9.13	799.2056	Iso/vitexin-*O*-(*O*-sinapoyl) hexoside	---	C_38_H_40_O_19_	-3.7		
15.	9.24	739.1868	Iso/vitexin-*O*-(*O*-coumaroyl) hexoside	739, 593, 413, 293	C_36_H_36_O_17_	-0.82		^115^
16.	9.25	769.1984	Iso/vitexin-*O*-(*O*-feruloyl) hexoside	769, 593, 413, 293	C_37_H_38_O_18_	0.58		
17.	9.26	187.0970	Azelaic acid	173, 143, 125	C_9_H_16_O_4_	-0.18	Fatty acids	^76^
18.	9.94	713.4733	Unknown-*C*-glycoside	713, 677, 651, 533, 367	C_35_H_70_O_14_	6.4	Flavonoids	^116^
19.	11.32	201.1127	Octanedicarboxylic acid (sebacic acid)	183, 139, 111	C_10_H_18_O_4_	0.09	Fatty acids	^76^
20.	14.3	329.2335	Trihydroxy octadecenoic acid	329, 229, 211, 187, 171	C_18_H_34_O_5_	2.27	Hydroxy fatty acids	
21.	19.13	227.1653 [M + H]^+^	Dihydrovomifoliol^a^	---	C_13_H_22_O_3_	4.97	Megastigmane	^38^
22.	19.15	343.2121	Tetrahydroxy octadecynoic acid	---	C_18_H_32_O_6_	0.23	Hydroxy fatty acids	^76^
23.	19.58	311.1866	Hydroxy-dioxoheptadecenoic acid	267, 249, 223	C_17_H_28_O_5_	2.41		https://www.metabolomicsworkbench.org/
24.	21.11	277.1809	Oxo-heptadecatrienoic acid	233, 205	C_17_H_26_O_3_	1.91	Fatty acids	
25.	21.67	271.2274	Hydroxypalmitic acid	271, 253, 225, 211	C_16_H_32_O_3_	0.58	Hydroxy fatty acids	^38^
26.	23.1	367.1917	Quercetol B	367, 178	C_23_H_28_O_4_	0.54	Prenylated flavonoids	https://www.metabolomicsworkbench.org/
27.	25.21	205.1593	Di-tert-butylphenol	205, 189	C_14_H_22_O	0.58	Phenolics	^29,117^
28.	25.59	384.1820	Pentamethoxy tetrahydroprotoberberine	384, 194, 177	C_22_H_27_NO_5_	0.78	Alkaloids	^118^
29.	26.14	299.2018	Eicosahexaenoic acid	299	C_20_H_28_O_2_	2.32	Fatty acids	https://www.metabolomicsworkbench.org/
30.	26.2	381.0609 [M + H]^+^	Phelligridin D^a^	( + ): 381, 188, 116	C_20_H_12_O_8_	1.07	Isocoumarins	
31.	26.91	275.2004	Stearidonic acid	---	C_18_H_28_O_2_	−2.56	Fatty acids	^76^
32.	27.23	271.2271	Hydroxypalmitic acid isomer I	---	C_16_H_32_O_3_	−0.81		^38^
33.	28.71	277.2146	Linolenic acid	277, 259, 233, 215, 195	C_18_H_30_O_2_	−7.77		
34.	28.81	271.2274	Hydroxypalmitic acid isomer II	225, 211, 177, 117	C_16_H_32_O_3_	0.36		
35	30.71	279.2322	Linoleic Acid	279, 221, 133, 128, 103	C18H32O2	−0.74		
36.	32.94	255.2329	Palmitic acid	255, 237, 195, 149	C_16_H_32_O_2_	−0.39		^21^
37.	33.41	281.2483	Oleic acid	281, 237, 221, 181	C_18_H_34_O_2_	1.05		^38^
38.	35.13	310.3103 [M + H]^+^	Eicosenamide^a^	---	C_20_H_39_NO	−0.46	Fatty amides	

^a^Compounds detected in the positive ion mode

#### Flavonoids

Flavonoids represent a major class of phenolic compounds known for their health benefits mainly because of their antioxidant and anti-inflammatory properties. A total of 14 flavonoids were annotated, mainly as *C*- and *O*-glycosylated and acylated flavones. The studied melon peel extract showed 6 conjugates of luteolin (classified as 2 iso/orientin and its glycosides, 3 lucenin-II, and 1 chrysoeriol) and 6 conjugates of apigenin (derivatives of iso/vitexin). In addition, an unidentified *C*-glycoside and a prenylated flavan were detected.

#### Luteolin conjugates

These are *C*-glycosylated or *C*- and *O*-glycosylated and acylated luteolin derivatives. In the negative ion mode, they exhibited loss of water and extensive losses of 90 and 120 amu due to 0,3 and 0,2-cross ring cleavage of the sugar moiety, respectively, indicating *C*-glycosylation. A distinctive fragment ion of luteolin at *m/z* 285 was also detected in line with previous studies^38^. Peak **6** was identified as luteolin-*C*-hexoside (iso/orientin) based on its [M-H]^-^ at *m/z* 447.0938 and supported by the characteristic fragment ions *m/z* 429 [M-H-H_2_O], 357 [M-H-90]^-^, 327 [M-H-120]^-^, and 285. Luteolin-*C*-hexoside was previously isolated and identified in *C. sativus* and *C. melo*^38,76,77^.

Acylated luteolin glycosides were identified, such as iso/orientin-*O*-(*O*-coumaroyl) hexoside (peak **8**) which yielded the characteristic fragment ions *m/z* 635 [M-H-120]^-^, 609 due to loss of coumaroyl moiety [M-H-146]^-^, and 429 [M-H-146-180]^-^. The annotation of *O*-feruloyl-lucenin-II (peak **10**) was supported by the significant loss of 90 and 120 amu and the loss of feruloyl moiety (-176 amu) upon fragmentation; *m/z* 665 [M-H-120]^-^, 575 [M-H-120-90]^-^, 545 [M-H-120-120]^-^, 609 [M-H-176]^-^, and 489 [M-H-176-120]^-^^38^.

#### Apigenin conjugates

A total of 6 glycosylated and/or acylated apigenin derivatives were tentatively identified (**7,** **11**, and **13**-**16**). The loss of 162 amu indicates *O*-glycosylation, while loss of 90 and 120 and successive loss of 18 amu (water) confirm *C*-glycosylation. Based on their fragmentation pattern and the available literature, *C*- and *O*-glycosides of apigenin were annotated. Peak **7** was tentatively identified as isovitexin-*O*-hexoside, supported by the fragment ions *m/z* 431 of isovitexin [M-H-162]^-^, 413 [M-H-162-H_2_O]^-^, 311 [M-H-162-120]^-^, and 293 [M-H-162-H_2_O-120]^-^. Isovitexin-*O*-hexoside was previously identified in the leaves and fruits of *C. melo* and *C. sativus*^38,76^. Additionally, acylated isovitexin-*O*-hexosides were identified, e.g., caffeoyl, sinapoyl, coumaroyl, and feruloyl derivatives (**13**-**16**).

#### Fatty acids

A total of 15 fatty acids were tentatively identified in *C. melo* peel extract, primarily detected in the negative ionization mode. These comprised 2 dicarboxylic, 6 hydroxylated, 1 oxo-, 5 unsaturated, and a saturated fatty acid. These findings were summarized in Table 4. Five unsaturated fatty acids were annotated, including 4 polyunsaturated fatty acids (PUFA); eicosahexaenoic acid (**29**), stearidonic acid (**31**), linolenic acid (**33**), and linoleic acid (**35**) and 1 monounsaturated fatty acid (MUFA); oleic acid (**37**). Linolenic acid (Rt 28.71 min, *m/z* 277.2146) produced the fragment ions *m/z* 259, 233, due to loss of H_2_O and CO_2_, respectively, from the carboxylic acid group, 215 [M-H-C_2_H_6_O_2_]^-^, and 195 [M-H-C_6_H_10_]^-^, indicative of sequential cleavage along the polyunsaturated chain^38^.

The hydroxylated subclass included hydroxypalmitic acids (**25,** **32**, and **34**), trihydroxyoctadecenoic acid (**20**), and tetrahydroxyoctadecynoic acid (**22**). Hydroxypalmitic acid (*m/z* 271.227) and its isomers exhibited typical fragment ions *m/z* 253 [M-H-H_2_O]^-^, 225 [M-H-H_2_O-CO]^-^, and 211 [M-H- H_2_O-C_2_H_2_O]^-^ in agreement with the recent report^38^. One saturated fatty acid, palmitic acid (peak **36**), was identified. Upon fragmentation, the daughter ions *m/z* 237 and 195 are formed due to loss of water and C_2_H_4_O_2_, respectively. Oxo-heptadecatrienoic acid (peak **24**) is an oxygenated fatty acid. It showed fragment ions at *m/z* 233 [M-H-CO_2_]^-^ and 205 [M-H-CO_2_-CO]^-^, consistent with the presence of an oxo group. Two dicarboxylic fatty acids were tentatively identified. Azelaic acid (peak **17**) showed diagnostic fragments at *m/z* 143 and 125, indicative of decarboxylation and further dehydration. Similarly, octanedicarboxylic acid (peak **19**) displayed a deprotonated molecule at *m/z* 201.1127 which, upon fragmentation, yielded the fragment ions *m/z* 183, 139, and 111, corresponding to successive loss of H_2_O, CO_2_, and CO^76^.

#### Organic acids

Two organic acids were identified in *C. melo* peel extract; citric acid (**1**) and phenyllactic acid (**4**). Phenyllactic acid (*m/z* 165.0541) produced the characteristic fragment ions at *m/z* 147 [M-H-H_2_O]^-^, 119 [M-H-H_2_O-CO]^-^, and 103 [M-H-H_2_O-CO_2_]^-^, consistent with cleavage of its phenylpropanoid moiety^38^. Phenyllactic acid is known for its broad-spectrum antimicrobial activity^78^.

#### Megastigmanes

Megastigmanes are oxidative breakdown products of carotenoids, typically featuring a substituted cyclohexane structure and often contain hydroxyl or keto groups. This class is known to have antimicrobial, cytotoxic, and antioxidant activities^79^. In melon peel extract, dihydrovomifoliol (**21**) was identified as a key representative of this class detected in the positive ionization mode^38^.

The current study highlights the potential of melon peel as a functional ingredient, promoting resource valorization and enhancing the shelf-life and microbial quality of minced meat. The preservative effect of melon peel extract may be attributed, at least partly, to its richness in flavonoids which have been reported to display antimicrobial activity^72^. Luteolin and its *C*-glucoside iso/orientin, tentatively identified in melon peel extract in compounds **5,** **6**, and **8**-**10**, were reported to display antibacterial activity against resistant strains of *E. coli* by damaging the cell membrane, inducing ultrastructural deformation and leakage of bacterial contents, affecting ATP synthesis and riboflavin energy metabolism, downregulating the expression of various resistance genes, and inhibiting biofilm formation^80,81^. Vitexin, identified in compounds **7,** **11**, and **13**-**16**, was reported to reduce *S. aureus* pathogenesis by decreasing its surface hydrophobicity and interfering with biofilm formation^82^. Moreover, the peel extract is enriched in fatty acids which have been reported to prevent the growth of, or directly kill, bacteria, fungi, and other pathogens by affecting multiple cellular targets, including the cell membrane, and interfering with mechanisms of virulence, such as biofilm formation^83^. Future studies should focus on the isolation and functional validation of the individual compounds tentatively identified by LC-MS/MS. Identification of the most active constituents and their underlying preservation mechanisms is a prerequisite for developing standardized extracts for clean-label meat preservation.

### Greenness assessment

The adoption of green solvents and sustainable extraction methods has become an indispensable priority to meet environmental, economic and public health benchmarks. Green solvents used in food industry must have less or no toxicity, better biodegradability, and minimal environmental and energy footprint compared to conventional solvents. They should be obtained from renewable sources, be recyclable, and readily scalable to industrial applications. Bio-alcohols, terpenes, and deep eutectic solvents (DES) are among the most studied green solvents. Additionally, the adopted sample preparation methods must limit the use of hazardous chemicals, reduce wastes, and show favorable green profile from raw material extraction till end-of-life disposal^16^.

In the present work, fresh peel wastes were used without prior drying, which elevates energy and economic cost of the process, in line with previous studies^84^. Moreover, fresh peel was reported to display higher TPC compared to freeze-dried^73^ or oven-dried peel^85^. Ethanol was selected as an extracting solvent due to its relatively favorable sustainability profile and established use in green extraction. Although the ethanol employed in this study was not specifically obtained from the fermentation of renewable feedstocks, the emerging bio-ethanol industry is expected to become the major alcohol source in the future. Ethanol has wide solubility range of hydrophilic and lipophilic substances and its antimicrobial properties decrease contamination risks during extraction^86^.

Greenness evaluation was conducted according to AGREEprep metrics^87^. The ten individual steps of assessment in AGREEprep take scores ranging from 0 to 1, with the extremes representing the worst and ultimate performance, respectively. Each criterion has a default weight in the overall score. A score of 0 (red) was consistently assigned to C1 in all cases, attributable to both the laboratory-scale nature of the existing literature and the requirement for sample transportation prior to extraction. As methanol is considered toxic compared to ethanol or DES, its input score (C2) is assigned 0. C3 targets sustainable, reusable, and renewable materials, while C4 considered the waste generated throughout the extraction process, including toxic solvents (e.g., methanol). In the present work, ethanol can be regarded as sustainable^88^ and it was recycled, therefore its input score was 1 (green). C5 is in favor of minimal sample used during the extraction process. In all cases of waste treatment, an excess of material is submitted for pretreatment to ensure sufficient availability for extraction or for multiple experimental batches. The hourly sample extraction rate for C6 was estimated based on the overall extraction period including pre- and post-treatment processes. C7 refers to number of steps and degree of automation. Steps less than or equal to 2 with fully automated systems are assigned the highest score (green) and procedures with 6 steps or more using manual systems are assigned a score of 0 (red). The final score for C7 is the product of both sub-scores. C8 refers to the total estimated energy consumption and expressed in Wh/sample. It was assessed during the overall extraction process when equipment-specific data were available and scored 1 for <10 Wh/sample and zero for > 500 Wh/sample. C9 denotes the final determination technique which takes 0.75 score for all cases as they used the same standard spectrophotometric determination for TPC. C10 is attributed to operator’s safety, and its scoring reflected the number of identified hazards of chemicals.

The present work exhibited superior greenness compared to previously published studies describing the extraction of phenolics from melon peel using conventional solvents^73,89,90^ and DES^91^ as presented in Table 5. The detailed AGREEprep input scores are illustrated in Figs. S1–S3. Methanol, used by Sroy et al.^73^, is not considered safe for human consumption and its use for the extraction of phytochemicals in food industry must meet strict regulatory standards for maximum residue limits which entail additional costs^92^. The sample preparation method adopted by Rico et al (2021) involves multiple centrifugation steps and oven drying to obtain the water insoluble solid (WIS) which is then extracted by DES at high temperatures (80–90 °C) for longer time (up to 120 min). These conditions may compromise phenolics stability^85^, increase energy consumption, and decrease greenness score as shown in Table 5^91^. Moreover, ethanol, adopted in our work as a green solvent, often presents superiority regarding life-cycle assessment (LCA) compared to DES^15^, although such comparisons are system-dependent. The synthesis of DES components (e.g., urea, citric acid, and choline chloride) may involve hidden environmental burdens that can surpass the energy required for bio-ethanol production via fermentation^15^. DES are difficult to recover and recycle because of their very low vapor pressure. Furthermore, the recovery of extracted phytochemicals from DES system requires complex, energy-heavy, longer treatment steps (e.g., anti-solvent precipitation and solid phase extraction/ethanol desorption) which negate solvent greenness and drastically spike the economic cost of the process limiting its industrial-scale food applications^93^. Ethanol is a more practical, energy-efficient choice that can be readily transferable to large-scale extraction processes^48,94^. However, future studies should prioritize direct LCA and economic analysis comparisons of ethanol and different DES to enable a solid evaluation of their environmental impacts and industrial feasibility.

**Table 5:** Greenness assessment of the extraction procedure in comparison to literature

Sample treatment (solvent)	TPC	AGREEprep score	References
*Cucumis melo* L. var. *reticulatus*, fresh peel, 100% methanol, SSR^a^ = 1:2 w/v, ultra-turrax, centrifugation	0.14 ± 0.01 mg GAE/g fresh peel		^89^
	≈ 0.12 ± 0.04 mg GAE/g fresh peel		^90^
	2.45 ± 0.24 mg GAE/g fresh peel		^73^
*Cucumis melo* L. var. *piel de sapo*, dried peel, DES (sodium acetate/urea/water), SSR = 2–5%, w/w	4.35 mg GAE/g dried peel		^91^
*Cucumis melo* L. var. *cantalupensis*, fresh peel, 95% etanol, SSR = 1:2, w/v, UAE	2.72 ± 0.03 mg GAE/g fresh peel (98.00 ± 1.21 mg GAE/g dry extract)		Present work

^a^ SSR Solid to solvent ratio

After ethanol extraction, the exhausted plant residue (cellulose-rich solid fraction) was reused as a potential compost ingredient in a small-scale garden setting^18,19^. However, comprehensive analysis of its physicochemical parameters and soil nutrient availability was not performed and needs dedicated future investigations. Another potential downstream application is the use of post-extraction residue for bio-ethanol production, particularly following the removal of phenolics which are known inhibitors of enzymatic hydrolysis and fermentation^17^. However, these pathways were not evaluated in the present study.

The present work provides a multifunctional and sustainable opportunity for eco-conscious valorization of fruit waste in minced beef industry. Green extracts of melon and prickly pear peels, incorporated in minced beef, effectively reduced lipid oxidation and inhibited the growth of spoilage and pathogenic microorganisms, during refrigerated storage, without negatively affecting sensory acceptability. Fortified minced beef samples showed significantly lower pH, peroxide value, and total volatile basic nitrogen levels, suggesting less microbial and oxidative degradation concomitant with significant improvement in sensory quality (appearance, odor, consistency, texture, and overall acceptability) compared to unfortified minced beef. Shelf-life was extended up to 15 days in treated samples, whereas the control deteriorated by day 6. Melon peel was the most effective in preserving minced beef bacteriological quality, especially reducing *Enterobacteriaceae*, *Pseudomonas*, and staphylococci counts as well as eradicating *L. monocytogenes* in a targeted viability study. The broad antioxidant properties of melon peel and its rich phytochemical composition, tentatively suggested by LC-qTOF-MS/MS analysis, open the door for its valorization as a functional ingredient that capitalizes on food resources, reduces waste, and identifies novel sustainable additives. The implemented green extraction methodology achieved better greenness and a higher recovery of phenolic compounds than previously published conventional and recent extraction techniques. By pairing this simple, efficient extraction process with the reduction of agro-industrial waste, our approach merits development into a viable circular economy model. The low overall processing cost makes scale-up to an industrial level feasible, though this requires further economic investigation. Despite these promising findings, some limitations should be recognized. The study was performed using relatively high extract concentrations. The applicability of these levels in industrial scale should be further evaluated. Additionally, because the greenness of the extraction process was indirectly compared to published work, a comprehensive side-by-side sustainability assessment is recommended. Future research should focus on optimizing extract concentrations, validating the activity of individual compounds, and conducting techno-economic and life-cycle analyses to better evaluate industrial applicability.

## Methods

### Plant material and green extraction

Fresh melon (*Cucumis melo* L. var. *cantalupensis*, family Cucurbitaceae) seeds and peels and prickly pear (*Opuntia ficus-indica* (L.) Mill., family Cactaceae) peels were collected from local juice stores in July 2024, repurposing them as high-value secondary raw materials. They were taxonomically authenticated by Reem Samir Hamdy, Professor of Plant Taxonomy, Department of Botany, Faculty of Science, Cairo University, Egypt. Voucher specimens of melon (PHG-P-CM-534) and prickly pear (PHG-P-OF-545) were deposited at the herbarium of the Pharmacognosy Department, Faculty of Pharmacy, Ain Shams University, Cairo, Egypt. Plant materials were transferred in an icebox to the laboratory. For green sample preparation, fresh samples were homogenized in a standard blender (1 min, 200 Wh) to maximize the surface area available for mass transfer and stored at −5 °C for 48 h till extraction. The homogenized material (50 g) was mixed with 100 mL 95% ethanol (Fischer Scientific, UK) and subjected to ultrasound-assisted extraction (UAE) (10 min, 45 kHz, 150 Wh, Crest Tru-Sweep 690 HTAE, Trenton, NJ, USA). While previous studies reported the use of hydroalcoholic mixtures for the extraction of phenolics from dry peels^95^, other studies used absolute methanol or 95% ethanol for the extraction of phenolics, particularly from fresh peels^73–75,89,90^. Herein, the fruit wastes were used fresh without a drying step. Therefore, 95% ethanol was used to compensate for the higher moisture content in fresh waste. In addition, high ethanol content was preferred to limit the co-extraction of highly polar, non-target compounds such as sugars.

UAE is based on acoustic cavitation and low-temperature operation which is favored for thermosensitive compounds^14^. Cavitation improves solvent penetration power and mass transfer rates via its mechanical effects, facilitating the extraction of bioactive compounds from the plant matrix and decreasing the extraction time. However, prolonged cavitation can lead to structural breakdown and thermal degradation of extracted substances^14^. The sonication time was selected after literature review and to increase the number of samples processed per hour, which is advantageous in terms of greenness and industrial feasibility^96^. The overall extraction yield was found satisfactory under the selected conditions. Extracts were filtered (Whatman cellulose filter paper), evaporated till dryness using a rotary evaporator (R 3000 Büchi, Flawil, Switzerland), and kept at −5 °C till further analysis. Ethanol was recovered for recycling and the exhausted plant residue was sun-dried, resulting in a coarse particulate (0.3–1 cm). The dried residue was rehydrated and uniformly mixed with mature compost prior to small scale composting in a garden setting. However, comprehensive analysis of physicochemical parameters and soil nutrient availability was beyond the scope of this study and warrants dedicated future investigation.

### Minimum effective concentration determination by agar well diffusion assay

The tested reference bacterial strains of *L. monocytogenes* ATCC 7494, *B. cereus* 10876, *S. aureus* ATCC 25923, *P. aeruginosa* ATCC 27853 were obtained from the Dairy Department at the National Research Centre and Animal Health Research Institute (Dokki, Giza, Egypt) while the isolated and molecular identified strain of *E. coli*, *K. pneumonia* as well as the two fungal strains (the molecular identified *C. albicans* and *A. parasiticus*) were obtained from the Microbiology Department, Faculty of Veterinary Medicine, Cairo University. Microbial suspensions of about 10^5^ CFU/mL were prepared according to the method previously mentioned by Gadallah et al.^97^. Then, minimum effective concentration, MEC, values of the prepared concentrations of melon peel, seed, and prickly pear peel (10, 7.5, 5, 2.5, 1, 0.5, 0.25, 0.1% w/v) against the aforementioned strains were determined by the agar well diffusion method as described by Gadallah et al.^97^. Based on the results of MEC, the dried extracts were used separately and in combination in the following tests in a 10% (w/w) concentration.

### Assessment of the efficacy of melon and prickly pear peel extracts in improving microbiological, physicochemical and sensory qualities of beef minced meat

Fresh beef cuts were bought from a butcher shop in Cairo, Egypt, packed in sterile plastic bags, and transferred in an ice box to the Food Microbiology Laboratory, Department of Food Hygiene and Control, Faculty of Veterinary Medicine, Cairo University. The fresh beef was cut by a sterile, sharp knife and minced using a clean, sterilized 0.32 cm grinder plate (Seydelmann NW 114 E; Stuttgart, Deutschland, Germany). The prepared minced beef was divided into four treatments as follows: the first group was fortified with melon peel extract 10%, w/w (MP), the second group was fortified with prickly pear peel extract 10%, w/w (PP), the third one was fortified with a mixture of prickly pear and melon peel extracts in a concentration of 5%, w/w for each one (1:1) (PP + MP), while the fourth group was left as a negative control without any fortification (C-Ve). Following that, the prepared groups were refrigerated at 4 ± 1 °C for 15 days. Samples were examined on day zero (processing day), 3rd, 6th, 9th, 12th, and 15th days of the storage period or until deterioration of the prepared groups for the following analyses.

#### Microbiological analysis

The microbial quality of the prepared groups was evaluated by determining the aerobic plate count (APC), psychrotrophic count, *Pseudomonas* count, *Enterobacteriaceae* count, staphylococci, yeast, and mold counts according to the method described by the American Public Health Association^98^. Briefly, 10 g of minced beef were homogenized with 90 mL of sterile Ringer’s solution (Oxoid BR0052G, Hampshire, England) in a sterile homogenizing bag using a stomacher (Seward 80 Lab Blender, compact, 110VAC) for 2 min to achieve a 10^−1^ dilution. From this, a series of tenfold dilutions was conducted. The APC was determined by spreading 0.1 mL from the diluted samples onto the surface of two sets of Plate Count Agar (PCA, Oxoid CM0325B, Hampshire, England), followed by incubation at 35 °C for 48 h. Another set of inoculated PCA was incubated at 7 °C for 10 days to count psychrotrophic bacteria. *Enterobacteriaceae* and staphylococci counts were obtained after incubating the inoculated Violet Red Bile Glucose agar and Baird-Parker plates (Oxoid, Hampshire, England) at 37 °C for 24 h. Meanwhile, yeast, mold, and *Pseudomonas* counts were quantified by incubating the inoculated Sabaroud Dextrose Agar plates (SDA, Oxoid CM0041, Hampshire, England) and *Pseudomonas* agar base at 25 °C for 3–5 days.

#### Physicochemical examination

##### Determination of pH

According to the method described by Lee and Shin^99^, pH value was evaluated after stomaching of 5 g of minced beef from each treatment with 20 mL of distilled water and measured separately using a previously calibrated digital pH meter (Lovibond, Senso Direct).

#### Determination of peroxide value

Following the AOAC guidelines^100^, the samples (10 g) were placed in open beakers (250 mL volume) and kept in a ventilated thermostat. About 1 g of the meat samples was extracted from the beakers, precisely weighed, and dissolved in 25 mL mixture of chloroform and acetic acid (3:2). Subsequently, 0.5 mL of saturated potassium iodide solution in distilled water was added. The samples were agitated for one minute, and then 25 mL of distilled water was introduced. The released iodine was titrated with a 0.01 M sodium thiosulfate solution, using a 1% starch solution as an indicator. Peroxide value, PV (meq/kg meat), was calculated using Eq. 1:

1 $$\mathrm{Peroxide}\,\mathrm{value}=0.01\,\mathrm{xN}\,{\rm{x}}\,1000\,/{\rm{m}}$$

where N represents the volume of sodium thiosulfate used in the titration of a sample, measured in mL, and m denotes the mass of the meat sample in g. These measurements were taken in triplicate over a span of 15 days.

#### Determination of total volatile basic nitrogen

The total volatile basic nitrogen (TVB-N) of all examined samples was estimated following a macro-Kjeldahl distillation procedures outlined by Kearsley et al.^101^. Ten grams of minced beef were mixed with 300 mL tap water in a distilling flask, then 2 g magnesium oxide were added. A macro-Kjeldahl distillation apparatus was connected to the distillation flask containing 25 mL of 2% boric acid solution and few drops of methyl-red indicator, with distillation continued till obtaining 200 mL of distillate in exactly 10 min and the distillation continued for 25 min using the same heating rate. The distillate was titrated with 0.05 M (0.1 N) sulphuric acid until a faint pink color was obtained. The TVB-N (mg/100 g sample) was calculated according to Eq. 2:

2 $$\mathrm{TVB}-{\rm{N}}\,(\mathrm{mg}/100\,{\rm{g}}\,\mathrm{sample})=\mathrm{mL}\,\mathrm{of}\,\mathrm{titrant}\,(0.1\,{\rm{N}}\,\mathrm{sulphuric}\,\mathrm{acid})\times 14$$

(14: molecular weight of Nitrogen)

#### Proximate chemical composition

The treated and control groups of minced beef were analyzed for moisture by hot air oven (Heraeus Functionline T6), crude fat by Soxhlet apparatus, crude protein using Kjeldahal method (VELP Scintifica digester (DK 6)), and ash content using muffle furnace (Thermo Scientific™ FD1530M) using the procedures outlined by the Association of Analytical Chemists^100^. The carbohydrate percentages were calculated as 100 - (moisture% + fat% + protein% + ash%).

#### Sensory analysis

Sensory evaluation of raw and cooked minced beef from all groups was conducted according to ISO 8589:2007 standards in a specially designed and equipped sensory analysis room. Twelve well-trained assessors of both sexes, aged 25–45 years old, including staff members and postgraduate researchers from the Department of Food Hygiene and Control, Faculty of Veterinary Medicine, Cairo University, were chosen to perform the evaluation based on their prior experience in sensory evaluation of meat and meat products. Before the evaluation sessions, panelists received training sessions on the evaluation protocol, including clarification of sensory attributes and use of the scoring system following ISO-11035:1994 guidelines^102^. All panelists were asked to assess the raw sensory attributes for appearance, texture, consistency, odor, and overall acceptability, while for cooked minced beef samples, the evaluated attributes included color, flavor, tenderness, juiciness, and overall acceptability^103,104^. Sensory evaluation of cooked samples was carried out by cooking meat samples from all groups after forming them into uniform patties (approximately 50 g in weight and 1.5 cm thick) using a standardized stainless-steel mold to ensure consistency in size and shape. Then cooking was carried out at 180 °C in a hot air oven (HAEUS D63,450 Hanau, Germany) to a core temperature 75 °C. The cooking temperature was monitored by a probe (Hanna HI 985081-1; Pasadena, TX, USA). All samples were cooked under identical conditions to minimize variability. No additional fat or seasoning was added during cooking to avoid interference with sensory attributes. After cooking, samples were allowed to rest for 2 min, then cut into uniform portions and immediately served warm (approximately 50–55 °C) to the panelists in coded containers. Randomized and coded samples (three-digit codes) were presented individually to panelists to minimize bias. Evaluations were conducted under controlled environmental conditions using simulated daylight illumination at ambient temperature. Moreover, the cooked and raw sensory evaluations were performed at the same time during the same sessions and ranked each sensory parameter using a structured sensory evaluation form based on a 1 - 9-point hedonic scale, where 1 = “unacceptable”, 5 = “neither unacceptable nor acceptable”, and 9 = “extremely acceptable”. Scores ≥6 were considered indicative of acceptable quality. Assessors were asked to evaluate the samples based on overall acceptability (consumer-oriented perception). All evaluations were conducted in three replicates under the same condition.

### Impact of melon and prickly pear peel extracts on viability of *L. monocytogenes* in minced beef

The surface of the prepared minced beef meat was superficially sterilized with UV light under UV Cabinet (Cole-Parmer 9818 Series-Darkroom) for 15 min before testing, following the method reported by El-Sherif et al.^105^. Aseptic minced beef was inoculated with a prepared suspension of *L. monocytogenes* to obtain a final concentration of 3.01 log_10_ CFU/g. Then the inoculated beef was divided into four treatments as follows: the first group was fortified with melon peel (MP) extract 10%, w/w, the second group was fortified with prickly pear peel (PP) extract 10%, w/w, the third one was fortified with a mixture of MP and PP extracts in a concentration of 5%, w/w each (1:1), while the fourth group was left as a positive control without fortification (C+Ve). The prepared groups were stored at a refrigerated temperature of 4 ± 1 °C for 15 days. The counts of *L. monocytogenes* were estimated on day zero (processing day), 3rd, 6th, 9th, 12th, and 15th days of the storage period or until disappearance of the inoculated strain (absence of growth on the surface of Palcam agar from food homogenate ~ count is less than 10 CFU/g) according to the method described by American Public Health Association^98^. Briefly, tenfold serial dilutions of minced beef from the different treatments were prepared, then 1 mL from the diluted samples was poured onto sterile plates followed by adding Palcam agar (40–45 °C) (Oxoid, Hampshire, England) then incubation at 37 °C for 24–48 h.

### Assessment of the total phenolic (TPC) and total flavonoid contents (TFC) in melon peel extract

The total phenolic content (TPC) was determined using the Folin–Ciocalteu colorimetric method^106^. Folin-Ciocalteau reagent (3 mL, 10%, w/v, Oxford lab chem., India) was mixed with 0.05 mL of the tested extract and 0.8 mL aqueous sodium bicarbonate (7.5%, w/v, El-Nasr pharmaceutical chemicals, Egypt). The reaction solution was incubated at room temperature for 30 min and the absorbance was measured at 765 nm using UV/Vis spectrophotometer (Milton Roy, Spectronic 1201, SpectraLab, Canada). A calibration curve of gallic acid (Sigma-Aldrich, Darmstadt, Germany) was prepared and the results were expressed as mg gallic acid equivalent (GAE)/g extract.

The total flavonoid content (TFC) was determined spectrophotometrically according to the method described by Chang et al.^107^. Briefly, 0.1 mL of tested extract was mixed with 3.9 mL distilled water and 0.3 mL aqueous sodium nitrite (5%, w/v, El-Nasr pharmaceutical chemicals) solution. The mixture was allowed to react for 5 min then 0.3 mL of aqueous aluminum chloride (10%, w/v, El-Nasr pharmaceutical chemicals, Egypt) solution was added and allowed to react for another 6 min. The solution was treated with 2 mL of 1 mM sodium hydroxide and 2.4 mL distilled water and the absorbance readings were recorded at 510 nm against a sample blank using UV/Vis spectrophotometer (Milton Roy, Spectronic 1201). TFC was determined using a standard curve with quercetin (Sigma-Aldrich, Darmstadt, Germany) and the results were expressed as mg quercetin equivalents (QE)/g extract.

### LC-qTOF-MS/MS chemical profiling of melon peel extract

Chemical profiling of melon peel extract was carried out using an Agilent Technologies 6530 series qTOF LC-MS system equipped with an autosampler (G7129A), a column compartment (G7116A), and a quaternary pump (G7104C). A 5 µL injection volume was used with a Zorbax^®^ SB C-18 column, 2.7 μm, 150 mm × 3 mm (Agilent Technologies, USA). The mobile phase consisted of two solvents: Solvent A (aqueous formic acid, 0.1% v/v) and Solvent B (acetonitrile acidified with 0.1% formic acid), delivered at a flow rate of 0.3 mL/min. A gradient elution was applied as follows: 90% Solvent A for the first 2 min, 80% solvent A at 10 min, and 100% solvent B from 52 to 80 min. All solvents were HPLC grade and were purchased from Sigma-Aldrich (Darmstadt, Germany)

Mass spectra were recorded using electrospray ionization (ESI) in negative and positive ionization modes, with a capillary voltage of 4500 V. Mass spectra were acquired under a gas temperature of 200 °C, a drying gas flow rate of 8 L/min, and a nebulization pressure of 58 psi. The mass spectra ranged from *m/z* 50 to 3000 and the voltages for the skimmer and fragmentation were set at 65 V and 120 V, respectively, with a collision energy of 10 V. Mass data were processed using the Mass Hunter workstation B.06.00 (Agilent Technologies) and compounds were tentatively identified by comparing their mass spectra, exact mass, and fragmentation patterns to previously reported data.

### DPPH radical scavenging assay

The DPPH (2,2-diphenyl-1-picrylhydrazyl) free radical scavenging activity was assessed following the standard method^108^ with slight modifications. A freshly prepared 0.004% (w/v) alcoholic solution of DPPH (Sigma-Aldrich, Darmstadt, Germany) was stored in the dark at 10 °C. An alcoholic solution of the test extract was prepared separately. An aliquot of 40 μL of the test solution was added to 3 mL of DPPH solution. The absorbance was recorded immediately and monitored at 515 nm using a UV/Visible spectrophotometer (Milton Roy, Spectronic 1201). Absorbance readings were taken at 1-min interval till stabilization (typically 16 min). The decrease in absorbance indicated the scavenging of DPPH radicals by antioxidants present in the extract. Control samples (DPPH solution without extract) and a reference standard (ascorbic acid, Sigma-Aldrich, Darmstadt, Germany) were also measured under the same conditions. Each experiment was conducted in triplicate.

The inhibition percentage of DPPH radical was calculated using Eq. 3:

3 $$\mathrm{Inhibition} \% =[({{\rm{A}}}_{{\rm{c}}}-\,{{\rm{A}}}_{{\rm{T}}})/{{\rm{A}}}_{{\rm{c}}}\,{\rm{x}}\,100]$$

where A_C_ is the absorbance of the control at 0 min and A_T_ is the absorbance of the sample and DPPH at 16 min.

The IC₅₀ value, representing the concentration of the extract required to inhibit 50% of the DPPH radicals, was determined by plotting a dose-response curve using GraphPad Prism software (San Diego, CA, USA).

### ABTS radical scavenging assay

The ABTS [2,2′-azino-bis(3-ethylbenzothiazoline-6-sulfonic acid)] radical scavenging capacity was evaluated using the decolorization assay method described by Sánchez et al.^109^. The assay measured the sample’s ability to scavenge the ABTS^•+^ radical cation, which results in a reduction in absorbance. The ABTS^•+^ radical cation was generated by mixing 1.8 mM ABTS stock solution (Sigma-Aldrich, Darmstadt, Germany) with 0.63 mM potassium persulfate. The mixture was kept in the dark at room temperature for 12–16 h to ensure complete radical formation. Before use, the solution was diluted with ethanol until the absorbance reached 0.700 ± 0.030 at 734 nm. Dry extract samples were diluted at 1:10 ratio with 80% ethanol. Then, 190 μL of the ABTS^•+^ solution was added to 10 μL of the diluted extract in a microtiter plate. The absorbance at 734 nm was measured every minute for 13 min using a spectrophotometer at ambient temperature. Appropriate solvent blanks were included in each run. Ascorbic acid was served as the positive control, while ethanol was used as the negative control. All assays were performed in triplicate, and the results were averaged. The percentage of ABTS^•+^ radical scavenging activity was calculated using Eq. 4:

4 $$\mathrm{Free}\,\mathrm{radical}\,\mathrm{scavenging}\,\mathrm{activity}\, \% =[({{\rm{A}}}_{{\rm{n}}}-{{\rm{A}}}_{{\rm{s}}})\,{\rm{x}}\,100]\,/{{\rm{A}}}_{{\rm{n}}}$$

where A_n_ was the final absorbance of the negative control and A_s_ was the final absorbance of the sample.

### Ferric reducing antioxidant power (FRAP) assay

The FRAP assay was conducted to evaluate the reducing power of the extract, which reflects its potential antioxidant capacity. The method was based on the procedures described by Ferreira et al.^110^ which rely on the reduction of ferricyanide to ferrocyanide in the presence of antioxidants. A 1 mg/mL alcoholic solution of the extract (1 mL) was mixed with 2.5 mL of 0.2 M sodium phosphate buffer (pH 6.6) and 2.5 mL of 1% (w/v) potassium ferricyanide (Oxford lab chem., India). The mixture was incubated at 50 °C for 20 min. After incubation, 2.5 mL of 10% (w/v) trichloroacetic acid (Oxford lab chem., India) was added to stop the reaction. The mixture was then centrifuged at 1000 × *g* for 10 min. An aliquot of 2.5 mL from the supernatant was mixed with 2.5 mL of deionized water and 0.5 mL of freshly prepared 0.1% (w/v) ferric chloride solution. The absorbance of the resulting solution was measured at 700 nm against a blank using a spectrophotometer (Milton Roy, Spectronic 1201).

The increase in absorbance indicated a higher reducing power of the sample. The percentage of reducing capability was calculated using Eq. 5:

5 $$\mathrm{Reducing}\,\mathrm{capability}( \% )=100-[({{\rm{A}}}_{0}-{{\rm{A}}}_{{\rm{s}}})/{{\rm{A}}}_{0}\times 100]$$

where A₀ was the absorbance of the control and A_s_ was the absorbance of the sample. The IC₅₀ value, representing the concentration of extract required to achieve 50% reducing activity, was determined from dose-response curves plotted using GraphPad Prism software (San Diego, CA, USA).

### Statistical analysis

All experiments were conducted in triplicates. Results were expressed as mean ± SD using the Statistical Package for Social Science (SPSS Inc., Chicago, IL, USA), version 26. Comparison between the tested groups was performed using two-way analysis of variance (ANOVA) followed by applying LSD post-hoc test to determine the significant pair(s). Significance was set at *P* < 0.05.

## Supplementary information

Supplementary information

## Data Availability

No datasets were generated or analysed during the current study.

## References

[1] Bytyqi, H., Barros, A. N., Krauter, V., Smaoui, S. & Varzakas, T. Innovative preservation technologies and supply chain optimization for reducing meat loss and waste: current advances, challenges, and future perspectives. *Sustainability* **18**, 530, 10.3390/su18010530 (2026).

[2] Ben Hsouna, A., Ben Halima, N., Smaoui, S. & Hamdi, N. *Citrus lemon* essential oil: chemical composition, antioxidant and antimicrobial activities with its preservative effect against Listeria monocytogenes inoculated in minced beef meat. *Lipids Health Dis.* **16**, 146, 10.1186/s12944-017-0487-5 (2017). 28774297 10.1186/s12944-017-0487-5 PMC5543433

[3] Domínguez, R. et al. A comprehensive review on lipid oxidation in meat and meat products. *Antioxidants* **8**, 429, 10.3390/antiox8100429 (2019). 31557858 10.3390/antiox8100429 PMC6827023

[4] Piper, P. W. Potential safety issues surrounding the use of benzoate preservatives. *Beverages* **4**, 33, 10.3390/beverages4020033 (2018).

[5] Chaturvedi, P. et al. Prevalence and hazardous impact of pharmaceutical and personal care products and antibiotics in environment: a review on emerging contaminants. *Environ. Res.* **194**, 110664. 10.1016/j.envres.2020.110664 (2021). 33400949 10.1016/j.envres.2020.110664

[6] Estévez, M. Critical overview of the use of plant antioxidants in the meat industry: opportunities, innovative applications and future perspectives. *Meat Sci.* **181**, 108610. 10.1016/j.meatsci.2021.108610 (2021). 34147961 10.1016/j.meatsci.2021.108610

[7] Arora, N. K. & Mishra, I. United Nations sustainable development goals 2030 and environmental sustainability: race against time. *Environ. Sustain.* **2**, 339–342, 10.1007/s42398-019-00092-y (2019).

[8] Awadallah, E., Khalil, N., Abulfadl, Y. S., Ibrahim, N. & Ayoub, I. M. Unleashing the power of golden berry leaves to counteract cyclophosphamide’s toll with antioxidant, anti-inflammatory, anti-apoptotic, and neurotransmitter boosting effects. *J. Ethnopharmacol.* **338**, 119114. 10.1016/j.jep.2024.119114 (2025). 39551281 10.1016/j.jep.2024.119114

[9] El-Nashar, H. A. et al. Neuroprotective effect of artichoke-based nanoformulation in sporadic Alzheimer’s disease mouse model: focus on antioxidant, anti-inflammatory, and amyloidogenic pathways. *Pharmaceuticals* **15**, 1202, 10.3390/ph15101202 (2022). 36297313 10.3390/ph15101202 PMC9610800

[10] Onyeaka, H. et al. Artificial intelligence in food system: innovative approach to minimizing food spoilage and food waste. *J. Agric. Food Res.* **21**, 101895. 10.1016/j.jafr.2025.101895 (2025).

[11] Birania, S. et al. Advances in development of biodegradable food packaging material from agricultural and agro-industry waste. *J. Food Process Eng.* **45**, e13930. 10.1111/jfpe.13930 (2022).

[12] Ibrahim, N., Abbas, H., El-Sayed, N. S. & Gad, H. A. *Rosmarinus officinalis* L. hexane extract: phytochemical analysis, nanoencapsulation, and in silico, in vitro, and in vivo anti-photoaging potential evaluation. *Sci. Rep.* **12**, 13102. 10.1038/s41598-022-16592-7 (2022). 35907916 10.1038/s41598-022-16592-7 PMC9338973

[13] Rațu, R. N. et al. Application of agri-food by-products in the food industry. *Agriculture* **13**, 1559, 10.3390/agriculture13081559 (2023).

[14] Xu, R. et al. Ultrasound-assisted extraction with deep eutectic solvents for valorization of agro-food waste: a sustainable and eco-friendly approach. *Ultrason. Sonochem*. 107500; 10.1016/j.ultsonch.2025.107500 (2025). 10.1016/j.ultsonch.2025.107500 PMC12356026 40782515

[15] Zaib, Q., Eckelman, M. J., Yang, Y. & Kyung, D. Are deep eutectic solvents really green? A life-cycle perspective. *Green. Chem.* **24**, 7924–7930, 10.1039/D2GC01752K (2022).

[16] Maki, M. A. A. et al. Green solvents as a sustainable strategy to reduce the impact of hazardous waste on human health. *Discov. Chem.* **2**, 342, 10.1007/s44371-025-00450-2 (2025).

[17] Rico, X., Yanez, R. & Gullon, B. Evaluation of strategies for enhanced bioethanol production from melon peel waste. *Fuel* **334**, 126710. 10.1016/j.fuel.2022.126710 (2023).

[18] Wu, R., Chen, M., Qin, Y., Liu, S. & Li, X. Combined hydrothermal and biological treatments for valorization of fruit and vegetable waste into liquid organic fertilizer. *Environ. Res.* **221**, 115262. 10.1016/j.envres.2023.115262 (2023). 36639011 10.1016/j.envres.2023.115262

[19] Cannavo, P., Herbreteau, A., Juret, D., Martin, M. & Guénon, R. Short-term effects of food waste composts on physicochemical soil quality and horticultural crop production. *J. Plant Nutr. Soil Sci.* **188**, 31–44, 10.1002/jpln.202400188 (2025).

[20] Vella, F. M., Calandrelli, R., Cautela, D. & Laratta, B. Natural antioxidant potential of melon peels for fortified foods. *Foods* **12**, 2523, 10.3390/foods12132523 (2023). 37444261 10.3390/foods12132523 PMC10340261

[21] Gómez-García, R., Campos, D. A., Aguilar, C. N., Madureira, A. R. & Pintado, M. Valorization of melon fruit (*Cucumis melo* L.) by-products: phytochemical and biofunctional properties with emphasis on recent trends and advances. *Trends Food Sci. Technol.* **99**, 507–519, 10.1016/j.tifs.2020.03.033 (2020).

[22] Hassan, S. A. et al. Unlocking the potential of muskmelon seeds as sustainable ingredients for the food industry. *Food Chem. Adv.* **7**, 101008. 10.1016/j.focha.2025.101008 (2025).

[23] Zhang, G., Chatzifragkou, A., Charalampopoulos, D. & Rodriguez-Garcia, J. Effect of defatted melon seed residue on dough development and bread quality. *LWT* **183**, 114892. 10.1016/j.lwt.2023.114892 (2023).

[24] da Cunha, J. A. et al. From seed to flour: sowing sustainability in the use of cantaloupe melon residue (*Cucumis melo* L. var. *reticulatus*). *PloS ONE* **15**, e0219229, 10.1371/journal.pone.0219229 (2020). 31895921 10.1371/journal.pone.0219229 PMC6939897

[25] Sipango, N. et al. Prickly pear (*Opuntia* spp.) as an invasive species and a potential fodder resource for ruminant animals. *Sustainability* **14**, 3719, 10.3390/su14073719 (2022).

[26] Aziz, R. A. & Khorshed, M. Effect of feeding prickly pear on productive performance of Maghrabian dromedary camel. *IJMAE* **4**, 61–78, 10.21608/ijmae.2024.309749.1037 (2024).

[27] Moraga-Babiano, L. et al. A comprehensive review of the nutritional, functional, and technological potential of prickly pear (*Opuntia ficus-indica*) in food processing. *QASCF* **17**, 10.15586/qas.v17i3.1558 (2025).

[28] Das, S. Natural therapeutics for urinary tract infections-a review. *Futur J. Pharm. Sci.* **6**, 64, 10.1186/s43094-020-00086-2 (2020). 33215041 10.1186/s43094-020-00086-2 PMC7498302

[29] El-anssary, A. A., Raoof, G. F. A., Saleh, D. O. & El-Masry, H. M. Bioactivities, physicochemical parameters and GC/MS profiling of the fixed oil of *Cucumis melo* L seeds: A focus on anti-inflammatory, immunomodulatory, and antimicrobial activities. *J. Herbmed. Pharmacol.* **10**, 476–485, 10.34172/jhp.2021.55 (2021).

[30] Wahdan, O., Bassuony, N., El-Ghany, Z. & El-Chaghaby, G. Antioxidant activity, antibacterial screening, proximate composition and GC-mass spectrometry analysis of cantaloupe seeds. *J. Agr. Chem. Biotechnol.* **7**, 291–295, 10.21608/jacb.2016.41152 (2016).

[31] Ali, S. K. et al. Phytochemical screening and characterization of the antioxidant, anti-proliferative and antibacterial effects of different extracts of *Opuntia ficus-indica* peel. *J. King Saud. Univ. Sci.* **34**, 102216, 10.1016/j.jksus.2022.10221 (2022).

[32] Kharrat, N. et al. Synergistic effect of polysaccharides, betalain pigment and phenolic compounds of red prickly pear (*Opuntia stricta*) in the stabilization of salami. *Int. J. Biol. Macromol.* **111**, 561–568, 10.1016/j.ijbiomac.2018.01.025 (2018). 29329812 10.1016/j.ijbiomac.2018.01.025

[33] Scavo, A. et al. Antimicrobial activity of cultivated cardoon (*Cynara cardunculus* L. var. altilis DC.) leaf extracts against bacterial species of agricultural and food interest. *Ind. Crops Prod.* **129**, 206–211, 10.1016/j.indcrop.2018.12.005 (2019).

[34] Suriyaprom, S. et al. Antioxidants of fruit extracts as antimicrobial agents against pathogenic bacteria. *Antioxidants* **11**, 602, 10.3390/antiox11030602 (2022). 35326252 10.3390/antiox11030602 PMC8945554

[35] Das, A. K. et al. Application of pomegranate by-products in muscle foods: oxidative indices, colour stability, shelf life and health benefits. *Molecules* **26**, 467 (2021). 33477314 10.3390/molecules26020467 PMC7830841

[36] Elgadir, M. A., Alhudhaibi, A. M., Abdallah, E. M. & Adiletta, G. Plant-based preservation of meat: a critical narrative review of bioactive extracts, essential oils, and next-generation delivery systems. *Front. Sustain. Food Syst.* **9**, 1722227. 10.3389/fsufs.2025.1722227 (2025).

[37] El-Hawary, S., El-Tantawy, M., Rabeh, M. & Badr, W. Chemical composition and antimicrobial activity of volatile constituents of cladodes, fruits peel and fruits pulp from *Opuntia ficus indica* (L.) Mill. (prickly pear) growing in Egypt. *Egypt. J. Chem.* **64**, 437–444, 10.21608/ejchem.2020.21137.2260 (2021).

[38] El-Sayed, H. M. et al. Metabolomics analysis of *Cucumis melo* var. *flexuosus* organs in correlation to its anti-inflammatory activity aided by chemometrics. *J. Pharm. Biomed. Anal.* **252**, 116512. 10.1016/j.jpba.2024.116512 (2025). 39405783 10.1016/j.jpba.2024.116512

[39] Alexandre, E. M. C. et al. Emergent technologies for the extraction of antioxidants from prickly pear peel and their antimicrobial activity. *Foods* **10**, 570, 10.3390/foods10030570 (2021). 33803279 10.3390/foods10030570 PMC7999070

[40] Palmeri, R., Parafati, L., Restuccia, C. & Fallico, B. Application of prickly pear fruit extract to improve domestic shelf life, quality and microbial safety of sliced beef. *Food Chem. Toxicol.* **118**, 355–360, 10.1016/j.fct.2018.05.044 (2018). 29787849 10.1016/j.fct.2018.05.044

[41] Nichita, A. et al. Physicochemical, microbiological, and sensory evaluation of plant-based meat analogs supplemented with phenolic extracts from olive mill by-products. *Foods* **14**, 3347, 10.3390/foods14193347 (2025). 41097516 10.3390/foods14193347 PMC12523269

[42] Murshed, H. M. et al. Enhancing the quality and shelf life of chevon using orange peel extract. *Meat Res.* **3**, 1–9, 10.55002/mr.3.6.77 (2023).

[43] Sharma, S., Majumdar, R. K., Mehta, N. K. & Nirmal, N. P. Effects of pineapple peel ethanolic extract on the physicochemical and textural properties of Surimi prepared from silver carp (*Hypophthalmichthys molitrix*). *Foods* **11**, 3223, 10.3390/foods11203223 (2022). 37430972 10.3390/foods11203223 PMC9601345

[44] Abdel-Naeem, H. H. S. et al. Antioxidant and antibacterial effect of fruit peel powders in chicken patties. *Foods* **11**, 301, 10.3390/foods11030301 (2022). 35159453 10.3390/foods11030301 PMC8834443

[45] Falowo, A. B., Fayemi, P. O. & Muchenje, V. Natural antioxidants against lipid–protein oxidative deterioration in meat and meat products: a review. *Food Res. Int.* **64**, 171–181, 10.1016/j.foodres.2014.06.022 (2014). 30011637 10.1016/j.foodres.2014.06.022

[46] Abbas, F. et al. A comparative study of extraction optimization, antioxidant potential, and fatty acid composition of *Cucumis sativus* and *Cucumis melo*. *Int. J. Pharm. Integr. Health Sci.* **5**, 125–136, 10.56536/ijpihs.v5i2.169 (2024).

[47] Parafati, L., Restuccia, C., Palmeri, R., Fallico, B. & Arena, E. Impact of prickly pear extract on the quality parameters of beef burger patties after cooking. *Food Biosci.* **42**, 101146. 10.1016/j.fbio.2021.101146 (2021).

[48] Pateiro, M., Gómez-Salazar, J. A., Jaime-Patlán, M., Sosa-Morales, M. E. & Lorenzo, J. M. Plant extracts obtained with green solvents as natural antioxidants in fresh meat products. *Antioxidants* **10**, 181, 10.3390/antiox10020181 (2021). 33513904 10.3390/antiox10020181 PMC7912489

[49] Elhassaneen, Y., Elbassouny, G., Hassan, R. & Meharam, E. Application of natural extracts in beef meatballs to prevent chemical and bacteriological spoilage agents, and extend its storage life. *Am. J. Food Sci. Technol.* **11**, 118–130, 10.12691/ajfst-11-4-1 (2023).

[50] Le Bourvellec, C. & Renard, C. M. Interactions between polyphenols and macromolecules: Quantification methods and mechanisms. *Crit. Rev. Food Sci. Nutr.* **52**, 213–248, 10.1080/10408398.2010.499808 (2012). 22214442 10.1080/10408398.2010.499808

[51] Ghoneim, S. et al. Improving the shelf life and quality of minced beef by *Cassia glauca* leaf extracts during cold storage. *Processes* **11**, 240, 10.3390/pr11010240 (2023).

[52] Zhou, T. et al. Effect of natural plant extracts on the quality of meat products: a meta-analysis. *Food Mater. Res.* **3**, 15, 10.48130/FMR-2023-0015 (2023).

[53] Hughes, J., Oiseth, S., Purslow, P. & Warner, R. A structural approach to understanding the interactions between colour, water-holding capacity and tenderness. *Meat Sci.* **98**, 520–532, 10.1016/j.meatsci.2014.05.022 (2014). 25034451 10.1016/j.meatsci.2014.05.022

[54] Ou, K. -y et al. A review of the antimicrobial activity of natural phenolic compounds and their applications in meat and meat products. *Shipin Kexue/Food Sci.* **44**, 358–366, 10.7506/spkx1002-6630-20220511-135 (2023).

[55] Masoud, L. M. A., Abdel-Naeem, H. H. S. & Zaki, H. M. B. A. Physicochemical properties, fatty acid profile, nutritional quality indices, sensory quality, topographical structure, and electrophoretic pattern of beef tongue and heart variety meats. *J. Food Compos. Anal.* **146**, 107868. 10.1016/j.jfca.2025.107868 (2025).

[56] Augustyńska-Prejsnar, A., Kačániová, M., Hanus, P., Sokołowicz, Z. & Słowiński, M. Microbial and sensory quality changes in broiler chicken breast meat during refrigerated storage. *Foods* **13**, 4063, 10.3390/foods13244063 (2024). 39767005 10.3390/foods13244063 PMC11675927

[57] Silva, M. A. et al. Nutritional and bioactive profiling of *Cucumis melo* L. by-products: towards a circular food economy. *Molecules* **30**, 1287, 10.3390/molecules30061287 (2025). 40142061 10.3390/molecules30061287 PMC11944493

[58] Günal-Köroğlu, D., Yılmaz, H., Subasi, B. G. & Capanoglu, E. Protein oxidation: the effect of different preservation methods or phenolic additives during chilled and frozen storage of meat/meat products. *Food Res. Int.* **200**, 115378. 10.1016/j.foodres.2024.115378 (2025). 39779159 10.1016/j.foodres.2024.115378

[59] Kalogianni, A. I., Lazou, T., Bossis, I. & Gelasakis, A. I. Natural phenolic compounds for the control of oxidation, bacterial spoilage, and foodborne pathogens in meat. *Foods* **9**, 794, 10.3390/foods9060794 (2020). 32560249 10.3390/foods9060794 PMC7353591

[60] Kang, J., Kim, B. H. & Yoon, Y. Meat spoilage by bacteria: influencing factors, volatile compounds, and organoleptic alterations. *Food Sci. Anim. Resour.* **46**, 42, 10.1007/s44463-025-00025-w (2026). 41849039 10.1007/s44463-025-00025-w PMC12995093

[61] Hussain, A. et al. Physicochemical and phytochemical analysis of three melon fruit (canary melon, watermelon, and muskmelon) peels, and their valorization in biscuits development. *Front. Sustain. Food Syst.* **8**, 1444017. 10.3389/fsufs.2024.1444017 (2024).

[62] Zor, M., Erdemir, E., Fettahoğlu, K., Topdas, E. F. & Şengül, M. Physicochemical properties, volatile compound profile changes, and sensory evaluation of meatballs with the addition of different vegetable peels. *Ital. J. Food Sci.* **37**, 286–299, 10.15586/ijfs.v37i3.2911 (2025).

[63] Zamuz, S. et al. The role of phenolic compounds against *Listeria monocytogenes* in food. A review. *Trends Food Sci. Technol.* **110**, 385–392, 10.1016/j.tifs.2021.01.068 (2021).

[64] Wang, J., Liu, S., Liu, B., Niu, X. & Deng, X. Luteolin inhibits listeriolysin O translation by directly targeting the coding region of the hly mRNA. *Front. Microbiol.* **10**, 1496, 10.3389/fmicb.2019.01496 (2019). 31312194 10.3389/fmicb.2019.01496 PMC6614183

[65] Gemmell, C. T., Parreira, V. R. & Farber, J. M. Controlling *Listeria monocytogenes* growth and biofilm formation using flavonoids. *J. Food Prot.* **85**, 639–646, 10.4315/JFP-21-135 (2022). 34982818 10.4315/JFP-21-135

[66] Seo, Y.-H., Han, C.-H., Lee, J.-M., Choi, S.-M. & Moon, K.-D. Effects of *Opuntia ficus indica* extracts on inactivation of *Escherichia coli* O157:H7 and *Listeria monocytogenes* on fresh-cut apples. *J. Korean Soc. Food Sci. Nutr.* **41**, 1009–1013, 10.3746/jkfn.2012.41.7.1009 (2012).

[67] Munteanu, I. G. & Apetrei, C. Analytical methods used in determining antioxidant activity: a review. *Int. J. Mol. Sci.* **22**, 3380, 10.3390/ijms22073380 (2021). 33806141 10.3390/ijms22073380 PMC8037236

[68] Wakid, S. A. & binti Harun, H. A. Antioxidant activity of melon fruit peel extracts. *J. Acad.* **8**, 53–57 (2020).

[69] Morais, D. R. et al. Antioxidant activity, phenolics and UPLC–ESI (–)–MS of extracts from different tropical fruits parts and processed peels. *Food Res. Int.* **77**, 392–399, 10.1016/j.foodres.2015.08.036 (2015).

[70] Gopalasatheeskumar, K. & Kalaichelvan, V. Antioxidant potential of different parts (leaves, stem, fruit, seed, flower and root) extracts of *Cucumis melo* var *agrestis*. *Int. J. Pharm. Sci. Res.* **12**, 465–469, 10.13040/IJPSR.0975-8232.12(1).465-69 (2021).

[71] Leopoldini, M., Russo, N. & Toscano, M. The molecular basis of working mechanism of natural polyphenolic antioxidants. *Food Chem.* **125**, 288–306, 10.1016/j.foodchem.2010.08.012 (2011).

[72] Chen, X., Lan, W. & Xie, J. Natural phenolic compounds: antimicrobial properties, antimicrobial mechanisms, and potential utilization in the preservation of aquatic products. *Food Chem.* **440**, 138198. 10.1016/j.foodchem.2023.138198 (2024). 38128429 10.1016/j.foodchem.2023.138198

[73] Sroy, S., Miller, F. A., Fundo, J. F., Silva, C. L. & Brandão, T. R. Freeze-drying processes applied to melon peel: assessment of physicochemical attributes and intrinsic microflora survival during storage. *Foods* **11**, 1499, 10.3390/foods11101499 (2022). 35627069 10.3390/foods11101499 PMC9141695

[74] Mallek-Ayadi, S., Bahloul, N. & Kechaou, N. Characterization, phenolic compounds and functional properties of *Cucumis melo* L. peels. *Food Chem.* **221**, 1691–1697, 10.1016/j.foodchem.2016.10.117 (2017). 10.1016/j.foodchem.2016.10.117 27979149

[75] Vella, F. M., Cautela, D. & Laratta, B. Characterization of polyphenolic compounds in cantaloupe melon by-products. *Foods* **8**, 196, 10.3390/foods8060196 (2019). 31174393 10.3390/foods8060196 PMC6617032

[76] El-Akad, R. H., El-Din, M. G. S. & Farag, M. A. How does *Lagenaria siceraria* (bottle gourd) metabolome compare to *Cucumis sativus* (cucumber) F. Cucurbitaceae? A multiplex approach of HR-UPLC/MS/MS and GC/MS using molecular networking and chemometrics. *Foods* **12**, 771, 10.3390/foods12040771 (2023). 36832849 10.3390/foods12040771 PMC9956347

[77] Mukherjee, P. K., Nema, N. K., Maity, N. & Sarkar, B. K. Phytochemical and therapeutic potential of cucumber. *Fitoterapia* **84**, 227–236, 10.1016/j.fitote.2012.10.003 (2013). 23098877 10.1016/j.fitote.2012.10.003

[78] Rajanikar, R. et al. Phenyllactic acid: a green compound for food biopreservation. *Food Control* **128**, 108184. 10.1016/j.foodcont.2021.108184 (2021).

[79] Samra, R. M., Othman, A., Elsbaey, M., Amen, Y. & Shimizu, K. Comprehensive review on megastigmane glycosides: Sources, bioactivities, and 13C NMR spectroscopic data. *Phytochem. Lett.* **60**, 19–89, 10.1016/j.phytol.2024.01.008 (2024).

[80] Ding, Y. et al. Antibacterial activity and mechanism of luteolin isolated from *Lophatherum gracile* Brongn. against multidrug-resistant *Escherichia coli*. *Front. Pharmacol.* **15**, 1430564. 10.3389/fphar.2024.1430564 (2024). 38983919 10.3389/fphar.2024.1430564 PMC11232434

[81] Zuo, J. et al. Antimicrobial and antibiofilm activity of isoorientin against carbapenem non-sensitive *Escherichia coli* from raw milk of goats. *J. Anim. Sci.* **101**, skad047. 10.1093/jas/skad047 (2023). 36762933 10.1093/jas/skad047 PMC9985329

[82] Das, M. C. et al. Vitexin alters *Staphylococcus aureus* surface hydrophobicity to obstruct biofilm formation. *Microbiol. Res.* **263**, 127126. 10.1016/j.micres.2022.127126 (2022). 35914415 10.1016/j.micres.2022.127126

[83] Andrew, P. D. Potential applications of antimicrobial fatty acids in medicine, agriculture and other industries. *Recent Pat. Antiinfect. Drug Discov.* **7**, 111–122, 10.2174/157489112801619728 (2012). 22630821 10.2174/157489112801619728

[84] Talekar, S., Patti, A. F., Vijayraghavan, R. & Arora, A. An integrated green biorefinery approach towards simultaneous recovery of pectin and polyphenols coupled with bioethanol production from waste pomegranate peels. *Bioresour. Technol.* **266**, 322–334, 10.1016/j.biortech.2018.06.072 (2018). 29982054 10.1016/j.biortech.2018.06.072

[85] Abd Rahman, N. F., Shamsudin, R., Ismail, A., Shah, N. N. A. K. & Varith, J. Effects of drying methods on total phenolic contents and antioxidant capacity of the pomelo (*Citrus grandis* (L.) Osbeck) peels. *Innov. Food Sci. Emerg. Technol.* **50**, 217–225, 10.1016/j.ifset.2018.01.009 (2018).

[86] Lee, J.-E. et al. The influence of solvent choice on the extraction of bioactive compounds from Asteraceae: a comparative review. *Foods* **13**, 3151, 10.3390/foods13193151 (2024). 39410186 10.3390/foods13193151 PMC11475975

[87] Pena-Pereira, F., Tobiszewski, M., Wojnowski, W. & Psillakis, E. A tutorial on AGREEprep an analytical greenness metric for sample preparation. *Adv. Sample Prep.* **3**, 100025. 10.1016/j.sampre.2022.100025 (2022).

[88] Kumar, V. & Sinha, A. R. Sustainable ethanol production: CO_2_ emission analysis and feedstock strategies through life cycle assessment. *Energy Sustain. Dev.* **88**, 101775. 10.1016/j.esd.2025.101775 (2025).

[89] Fundo, J. F. et al. Physicochemical characteristics, bioactive compounds and antioxidant activity in juice, pulp, peel and seeds of cantaloupe melon. *J. Food Meas. Charact.* **12**, 292–300, 10.1007/s11694-017-9640-0 (2018).

[90] Miller, F. A., Fundo, J. F., Garcia, E., Silva, C. L. & Brandão, T. R. Effect of gaseous ozone process on cantaloupe melon peel: Assessment of quality and antilisterial indicators. *Foods* **10**, 727, 10.3390/foods10040727 (2021). 33808125 10.3390/foods10040727 PMC8066758

[91] Rico, X., Nuutinen, E.-M., Gullón, B., Pihlajaniemi, V. & Yáñez, R. Application of an eco-friendly sodium acetate/urea deep eutectic solvent in the valorization of melon by-products. *Food Bioprod. Process.* **130**, 216–228, 10.1016/j.fbp.2021.10.006 (2021).

[92] European Parliament and Council of the European Union Directive 2009/32/EC of the European Parliament and of the Council of 23 April 2009 on the approximation of the laws of the Member States on extraction solvents used in the production of foodstuffs and food ingredients. *J. Eur. Union L* **141**, 3–11 (2009).

[93] Rico, X., Gullón, B. & Yáñez, R. A comparative assessment on the recovery of pectin and phenolic fractions from aqueous and DES extracts obtained from melon peels. *Food Bioprocess Technol.* **15**, 1406–1421, 10.1007/s11947-022-02823-2 (2022).

[94] Awad, A. M. et al. Green extraction of bioactive compounds from plant biomass and their application in meat as natural antioxidant. *Antioxidants* **10**, 1465, 10.3390/antiox10091465 (2021). 34573097 10.3390/antiox10091465 PMC8466011

[95] Dimtsas, V. et al. Exploring varied (green) extraction methods to optimize galia melon peel antioxidant potential. *Separations* **11**, 135, 10.3390/separations11050135 (2024).

[96] Hosseini, S. S., Khodaiyan, F., Kazemi, M. & Najari, Z. Optimization and characterization of pectin extracted from sour orange peel by ultrasound assisted method. *Int. J. Biol. Macromol.* **125**, 621–629, 10.1016/j.ijbiomac.2018.12.096 (2019). 30543886 10.1016/j.ijbiomac.2018.12.096

[97] Gadallah, A. H., Hafez, R. S., Fahim, K. M. & Ahmed, L. I. Application of rosemary oil nano-emulsion as antimicrobial and antioxidant natural alternative in pasteurized cream and Karish cheese. *Int. J. Food Microbiol.* **422**, 110823. 10.1016/j.ijfoodmicro.2024.110823 (2024). 38991433 10.1016/j.ijfoodmicro.2024.110823

[98] Salfinger, Y. & Tortorello, M. L. *Compendium of Methods for the Microbiological Examination of Foods* (APHA Press, an imprint of American Public Health Association, 2015).

[99] Lee, E. J. & Shin, H. S. Development of a freshness indicator for monitoring the quality of beef during storage. *Food Sci. Biotechnol.* **28**, 1899–1906, 10.1007/s10068-019-00633-5 (2019). 31807364 10.1007/s10068-019-00633-5 PMC6859128

[100] AOAC International. *Official Methods of Analysis of AOAC International.* 18^th^ edn (AOAC International, Gaithersburg, MD, USA, 2005).

[101] Kearsley, M. W., El-Khatib, L. & Gunu, C. O. K. A. Rapid determination of total volatile nitrogen in fish and meat. *JAPA* **21**, 123–128 (1983).

[102] ISO. *Sensory Analysis—Identification and Selection of Descriptors for Establishing a Sensory Profile by a Multidimensional Approach*. ISO 11035: 1994 (International Organization for Standardization, 1994).

[103] Coggins, P. C. Attributes of muscle foods: color, texture, flavor. in *Handbook of Meat, Poultry and Seafood Quality* (ed. Nollet, L. M. L.) 89-100 (Blackwell Publishing, 2007).

[104] AMSA. *Research guidelines for cookery, sensory evaluation and instrumental tenderness measurements of fresh meat* (National Live Stock and Meat Board, 1995).

[105] Elsherif, W. M., Zayed, G. M. & Tolba, A. O. Antimicrobial activity of chitosan- edible films containing a combination of carvacrol and rosemary nano-emulsion against Salmonella enterica serovar Typhimurium and Listeria monocytogenes for ground meat. *Int. J. Food Microbiol.* **418**, 110713. 10.1016/j.ijfoodmicro.2024.110713 (2024). 38718617 10.1016/j.ijfoodmicro.2024.110713

[106] Singleton, V. L., Orthofer, R. & Lamuela-Raventós, R. M. Analysis of total phenols and other oxidation substrates and antioxidants by means of folin-ciocalteu reagent. *Methods Enzymol.* **299**, 152–178 (1999).

[107] Chang, C. C., Yang, M. H., Wen, H. M. & Chern, J. C. Estimation of total flavonoid content in propolis by two complementary colorimetric methods. *J. Food Drug Anal.* **10**, 178–182, 10.38212/2224-6614.2748 (2002).

[108] Al Zahrani, N. A., El-Shishtawy, R. M., Elaasser, M. M. & Asiri, A. M. Synthesis of novel chalcone-based phenothiazine derivatives as antioxidant and anticancer agents. *Molecules* **25**, 4566, 10.3390/molecules25194566 (2020). 33036301 10.3390/molecules25194566 PMC7583060

[109] Samaniego Sánchez, C. et al. Different radical scavenging tests in virgin olive oil and their relation to the total phenol content. *Anal. Chim. Acta* **593**, 103–107, 10.1016/j.aca.2007.04.037 (2007). 17531830 10.1016/j.aca.2007.04.037

[110] Ferreira, I. C. F. R., Baptista, P., Vilas-Boas, M. & Barros, L. Free-radical scavenging capacity and reducing power of wild edible mushrooms from northeast Portugal: Individual cap and stipe activity. *Food Chem.* **100**, 1511–1516, 10.1016/j.foodchem.2005.11.043 (2007).

[111] Jo, H. E., Son, S. Y. & Lee, C. H. Comparison of metabolome and functional properties of three Korean cucumber cultivars. *Front. Plant Sci.* **13**, 882120. 10.3389/fpls.2022.882120 (2022). 35498687 10.3389/fpls.2022.882120 PMC9051474

[112] Farag, M. A. et al. A comparative metabolomics approach for Egyptian mango fruits classification based on UV and UPLC/MS and in relation to its antioxidant effect. *Foods* **11**, 2127, 10.3390/foods11142127 (2022). 35885370 10.3390/foods11142127 PMC9318453

[113] Hosoya, T., Masuda, Y., Ohba, S. & Kumazawa, S. Component analysis of *Cucumis melo* L. leaves and their antioxidant activity. *Nat. Prod. Res.* **40**, 627–634, 10.1080/14786419.2024.2416508 (2024). 39412363 10.1080/14786419.2024.2416508

[114] Olennikov, D. et al. Composition and Antilipase Activity of C, O-glycosylflavones from Yellow-Skinned Varieties of *Cucumis sativus* L. (Cucurbitaceae). *Pharm. Chem. J.* **58**, 1705–1712, 10.1007/s11094-025-03328-w (2025).

[115] Abou-Zaid, M. M., Lombardo, D. A., Kite, G. C., Grayer, R. J. & Veitch, N. C. Acylated flavone C-glycosides from *Cucumis sativus*. *Phytochemistry* **58**, 167–172, 10.1016/s0031-9422(01)00156-x (2001). 11524127 10.1016/s0031-9422(01)00156-x

[116] Sukadeetad, K., Nakbanpote, W., Heinrich, M. & Nuengchamnong, N. Effect of drying methods and solvent extraction on the phenolic compounds of Gynura pseudochina (L.) DC. leaf extracts and their anti-psoriatic property. *Ind. Crops Prod.* **120**, 34–46, 10.1016/j.indcrop.2018.04.020 (2018).

[117] Zhao, F., Wang, P., Lucardi, R. D., Su, Z. & Li, S. Natural sources and bioactivities of 2, 4-di-tert-butylphenol and its analogs. *Toxins* **12**, 35, 10.3390/toxins12010035 (2020). 31935944 10.3390/toxins12010035 PMC7020479

[118] Barbosa-Filho, J., da-Cunha, E. V. L. & Gray, A. I. Alkaloids of the menispermaceae in *The Alkaloids*. *Chem. Biol.* **54**, 1–190 (2000).

