# Supplementary material for: Green valorization of melon and prickly pear byproducts for functional use in minced beef
Source: NPJ Sci Food. 2026 Jun 10;10:184. doi: 10.1038/s41538-026-00922-4 (PMC13253867; doi:10.1038/s41538-026-00922-4)
Supplement: Supplementary file 1 — Supplementary information [file 41538_2026_922_MOESM1_ESM.pdf]

Green Valorization of Melon and Prickly Pear Byproducts for Functional Use in Minced Beef

Nehal Ibrahim[a,#,*], Shaimaa Fayez[a,#,*], Salma H. Katary[b], Hany Ibrahim[c], Lamiaa Ibrahim Ahmed[d], Gehad A. Ezzat[d]

[a] Pharmacognosy Department, Faculty of Pharmacy, Ain Shams University, 11566 Cairo, Egypt.
[b] Faculty of Pharmacy, Ain Shams University, 11566 Cairo, Egypt.
[c] Pharmaceutical Analytical Chemistry Department, Faculty of Pharmacy, Egyptian Russian University, Badr City, 11829 Cairo, Egypt.
[d] Food Hygiene and Control Department, Faculty of Veterinary Medicine, Cairo University, Giza, Egypt.

[#] These authors contributed equally to this work.
[*] Correspondence: Nehal Ibrahim (nehal.sabry@pharma.asu.edu.eg) and Shaimaa Fayez (shaimaa_fayez@pharma.asu.edu.eg), Pharmacognosy Department, Faculty of Pharmacy, Ain Shams University, 11566 Cairo, Egypt.

List of contents

Figure S1. The input scores of AGREEprep of conventional methanol extraction.[1-3]

Figure S2. The input scores of AGREEprep of DES extraction.[4]

Figure S3. The input scores of AGREEprep of the current work.

AGREEprep

Analytical Greenness Metric
for Sample Preparation

18/04/2026 17:05:01

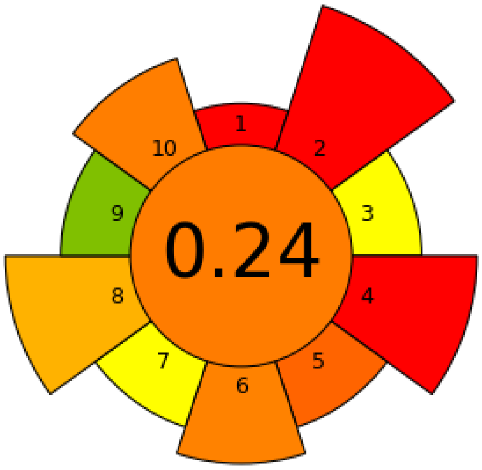

#	Criterion	Score	Weight
1.	**Sample preparation placement**<br>Sample preparation placement: Ex situ	0.0	1
2.	**Hazardous materials**<br>Mass [g] or volume [mL] of problematic materials: 50	0.0	5
3.	**Sustainability, renewability, and reusability of materials**<br>50-75% of reagents and materials are sustainable or renewable, but can only be used ONCE	0.5	2
4.	**Waste**<br>Mass [g] or volume [mL] of waste: 50	0.0	4
5.	**Size economy of the sample**<br>Mass [g] or volume [mL] of the sample: 25	0.2	2
6.	**Sample throughput**<br>Hourly sample throughput: 3	0.26	3
7.	**Integration and automation**<br>No. of sample prep. steps: 2 steps or fewer; degree if automation: Semi-automated systems	0.5	2
8.	**Energy consumption**<br>Approximate energy consumption per analysis [W]: 125	0.35	4
9.	**Post-sample preparation configuration for analysis**<br>Spectrophotometry, surface analysis techniques, voltammetry, potentiometry, etc.	0.75	2
10.	**Operator's safety**<br>No. of distinct hazards: 3 hazards	0.25	3

Figure S1. The input scores of AGREEprep of conventional methanol extraction.[1-3]

AGREEprep

Analytical Greenness Metric
for Sample Preparation

18/04/2026 21:57:01

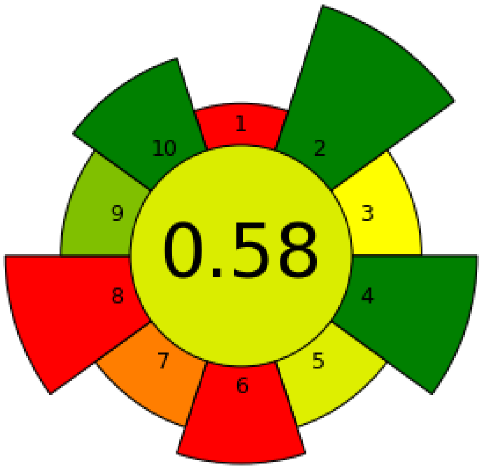

#	Criterion	Score	Weight
1.	**Sample preparation placement**<br>Sample preparation placement: Ex situ	0.0	1
2.	**Hazardous materials**<br>Mass [g] or volume [mL] of problematic materials: 0	1.0	5
3.	**Sustainability, renewability, and reusability of materials**<br>50-75% of reagents and materials are sustainable or renewable, but can only be used ONCE	0.5	2
4.	**Waste**<br>Mass [g] or volume [mL] of waste: 0	1.0	4
5.	**Size economy of the sample**<br>Mass [g] or volume [mL] of the sample: 2	0.57	2
6.	**Sample throughput**<br>Hourly sample throughput: 1	0.0	3
7.	**Integration and automation**<br>No. of sample prep. steps: 4 steps; degree if automation: Semi-automated systems	0.25	2
8.	**Energy consumption**<br>Approximate energy consumption per analysis [W]: 500	0.0	4
9.	**Post-sample preparation configuration for analysis**<br>Spectrophotometry, surface analysis techniques, voltammetry, potentiometry, etc.	0.75	2
10.	**Operator's safety**<br>No. of distinct hazards: No hazards or no exposure	1.0	3

Figure S2. The input scores of AGREEprep of DES extraction.

AGREEprep

Analytical Greenness Metric
for Sample Preparation

18/04/2026 23:43:01

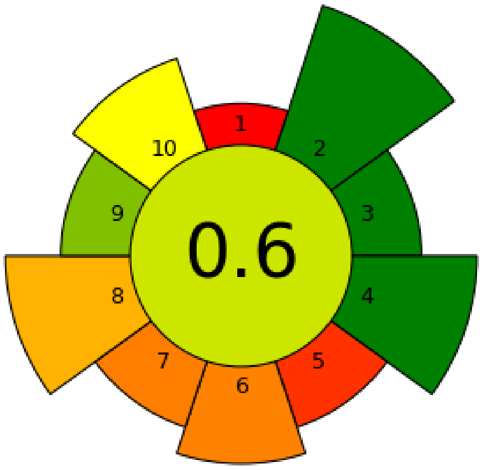

#	Criterion	Score	Weight
1.	**Sample preparation placement**<br>Sample preparation placement: Ex situ	0.0	1
2.	**Hazardous materials**<br>Mass [g] or volume [mL] of problematic materials: 0	1.0	5
3.	**Sustainability, renewability, and reusability of materials**<br>Only sustainable and renewable materials are used SEVERAL TIMES	1.0	2
4.	**Waste**<br>Mass [g] or volume [mL] of waste: 0	1.0	4
5.	**Size economy of the sample**<br>Mass [g] or volume [mL] of the sample: 50	0.1	2
6.	**Sample throughput**<br>Hourly sample throughput: 3	0.26	3
7.	**Integration and automation**<br>No. of sample prep. steps: 4 steps; degree if automation: Semi-automated systems	0.25	2
8.	**Energy consumption**<br>Approximate energy consumption per analysis [W]: 125	0.35	4
9.	**Post-sample preparation configuration for analysis**<br>Spectrophotometry, surface analysis techniques, voltammetry, potentiometry, etc.	0.75	2
10.	**Operator's safety**<br>No. of distinct hazards: 2 hazards	0.5	3

Figure S3. The input scores of AGREEprep of the current work.

References

1. Fundo, J. F. et al. Physicochemical characteristics, bioactive compounds and antioxidant activity in juice, pulp, peel and seeds of cantaloupe melon. *J. Food Meas. Charact.* **12,** 292-300; 10.1007/s11694-017-9640-0 (2018).
2. Miller, F.A., Fundo, J.F., Garcia, E., Silva, C.L. & Brandão, T.R. Effect of gaseous ozone process on cantaloupe melon peel: Assessment of quality and antilisterial indicators. *Foods* **10,** 727; 10.3390/foods10040727 (2021).
3. Sroy, S., Miller, F.A., Fundo, J.F., Silva, C.L. & Brandão, T.R. Freeze-drying processes applied to melon peel: Assessment of physicochemical attributes and intrinsic microflora survival during storage. *Foods* **11,** 1499; 10.3390/foods11101499 (2022).
4. Rico, X., Nuutinen, E.-M., Gullón, B., Pihlajaniemi, V. & Yáñez, R. Application of an eco-friendly sodium acetate/urea deep eutectic solvent in the valorization of melon by-products. Food Bioprod. Process. **130**, 216-228; 10.1016/j.fbp.2021.10.006 (2021).
